# Supplementary material for: Stress History Establishes a Transient Tolerant State That Shapes Antibiotic Survival Upon Resuscitation
Source: Adv Sci (Weinh). 2026 May 19;13(44):e21993. doi: 10.1002/advs.202521993 (PMC13336005; doi:10.1002/advs.202521993)
Supplement: Supplementary file 1 [file ADVS-13-e21993-s001.pdf]

Supporting information for: Stress History Establishes a Transient Tolerant State That Shapes Antibiotic Survival Upon Resuscitation

Kieran Abbott[1,†,‡], Georgeos Hardo[1,§], Ruizhe Li[†],
Jack Bradley[†], Ashraf Zarkan[‡], Somenath Bakshi[*,†]

[1]These two authors contributed equally
[†]Department of Engineering, University of Cambridge
[‡]Department of Genetics, University of Cambridge
[§]Department of Biology, United Arab Emirates University

[*]Correspondence to Somenath Bakshi: somenath.bakshi@eng.cam.ac.uk

Supplementary Figures

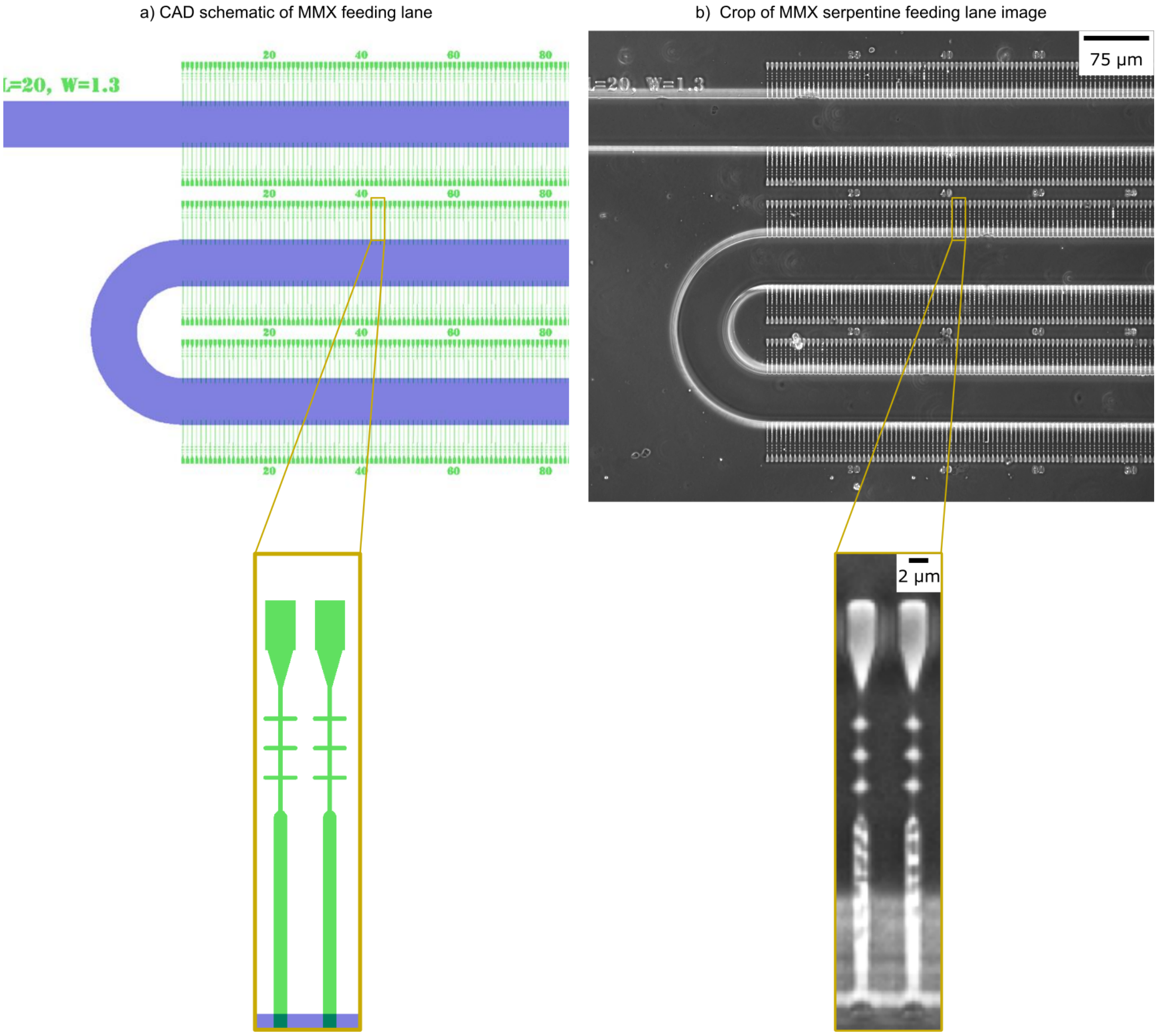

Figure S1: MMX flow lane and trench layout for high-throughput uniform treatment per lane. a) A CAD schematic showing the feeding lane (blue) and corresponding cell trenches (green), along with fiducial markers which allow us to identify the relative position of each trench in an FOV without knowing the absolute position of the microscope stage. The thickness of the feeding lane is 25 μm and 1.1 μm for cell trenches. **b)** A phase contrast image showing the PDMS cast MMX feeding lane and a zoom is shown below where bacterial cells are loaded in the trenches. The trench width is 1.3 μm, length is 20 μm, backport length is 12.5 μm, and width 0.4 μm, except the junctions. Finally, the evaporation chamber area is approximately 20.7 μm^2. The typical snake-like layout of flow lanes lead to weak loading of regular mother machine trench design, possibly related to evaporation from parallel lanes through the separating PDMS affecting each other. The new trench design with evaporation chambers in MMX significantly improves loading in such scenarios.

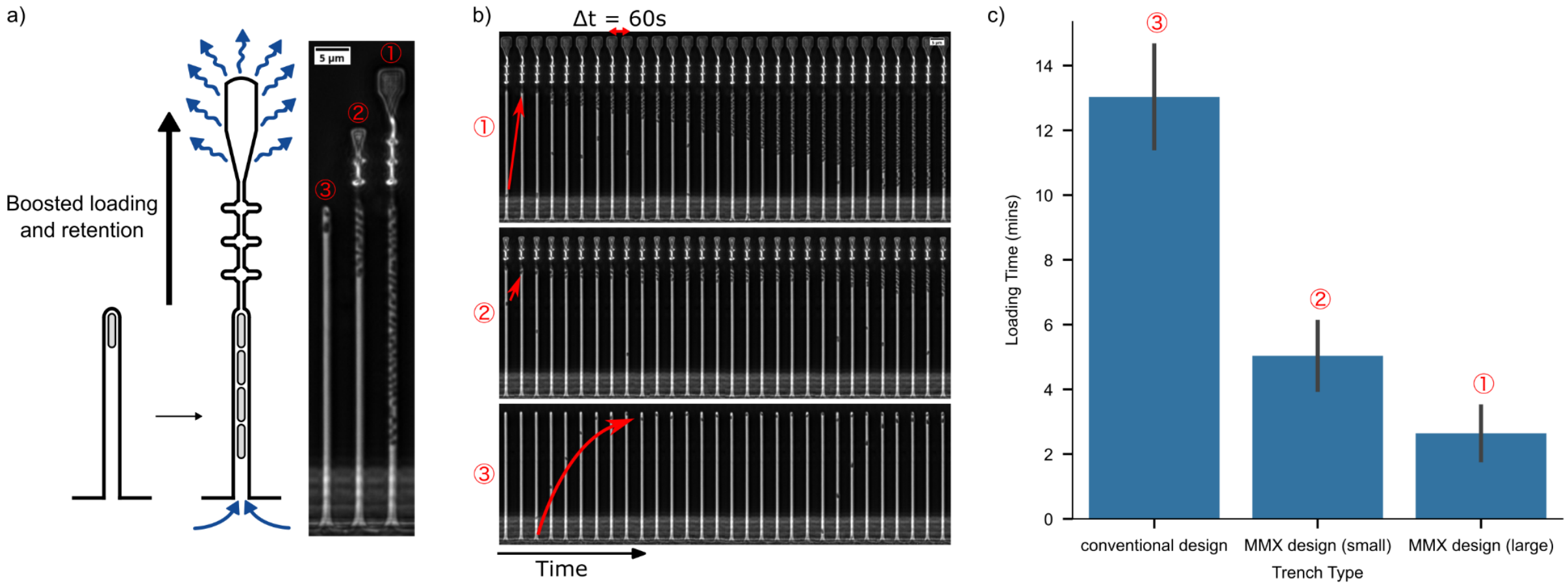

Figure S2: MMX trench design for evaporation-assisted efficient loading. a) The MMX trench design for evaporation assists efficient loading. A schematic cartoon is shown on the left. The real image (right) shows different designs benchmarked on the same test device and the same flow lane for direct comparison. **b)** Time-lapse imaging over a 30-minute period comparing loading dynamics in a MMX-design trench design (top), a MMX-design trench design with a smaller top chamber (middle), and a conventional mother machine trench (bottom). **c)** The bar plot shows the quantification of loading performance. Mean waiting times to obtain at least one cell per trench are 2.6 min (n = 40) for the MMX design, achieving ~100% occupancy within 30 min.

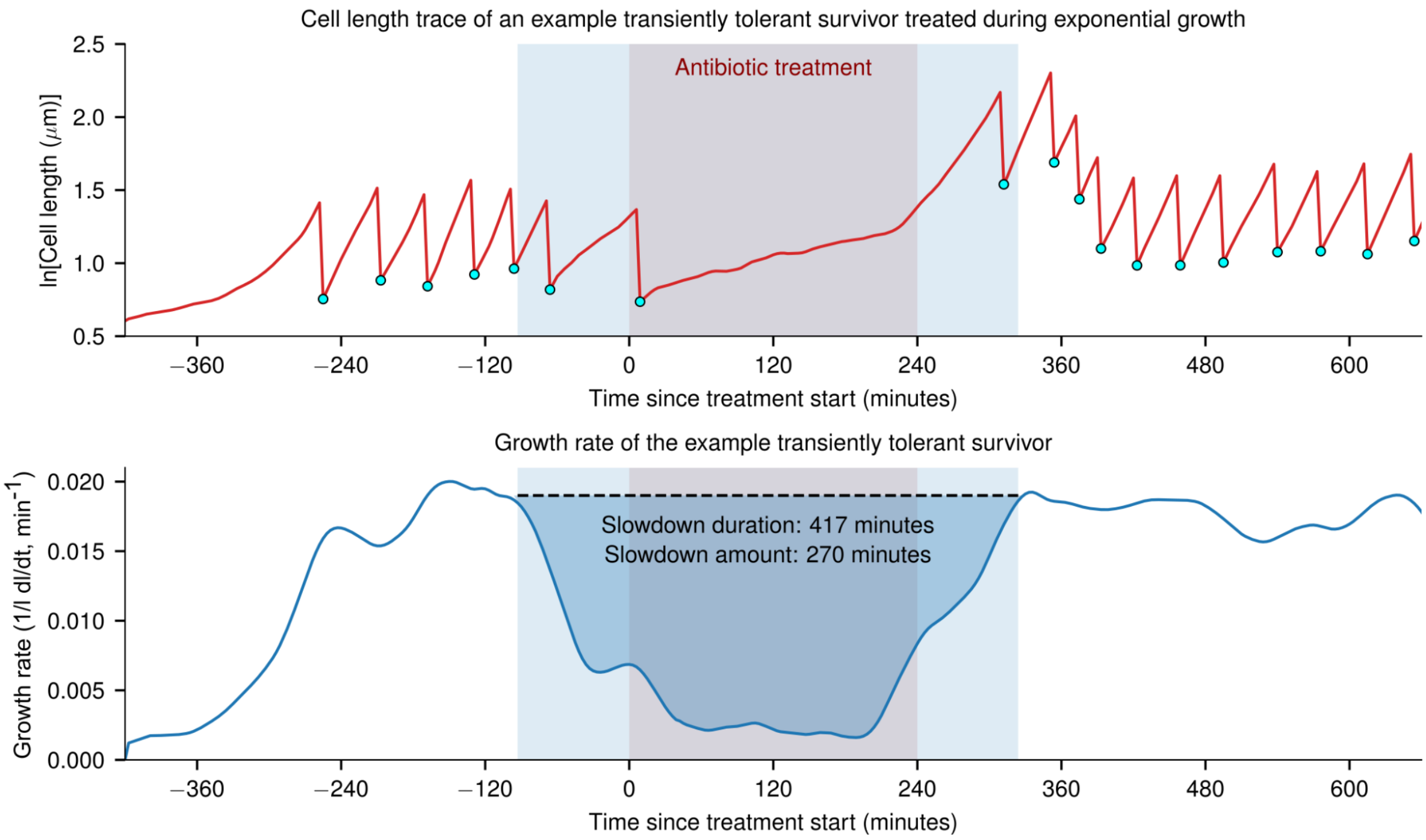

Figure S3: Exponential phase cells survive antibiotic exposure by spontaneous transient growth slowdown, not complete growth arrest. Plot of cell length against time (top) and corresponding plot of growth rate against time (bottom) for an example *E. coli* cell within an exponential phase population that survived 4 hours of treatment with 50 µg/mL ampicillin. A 24 hour old culture of cells was loaded into lanes of the MMX device and grown with fresh media for 7 hours prior to treatment start. Cyan circles indicate division events. Antibiotic treatment time is highlighted in red. The cell growth rate slowdown event is highlighted in blue. Slowdown duration corresponds to the time between the start and the end of the growth rate slowdown event. Slowdown amount refers to the total lag time accumulated during the slowdown event, using a similar definition to lag time in[1].

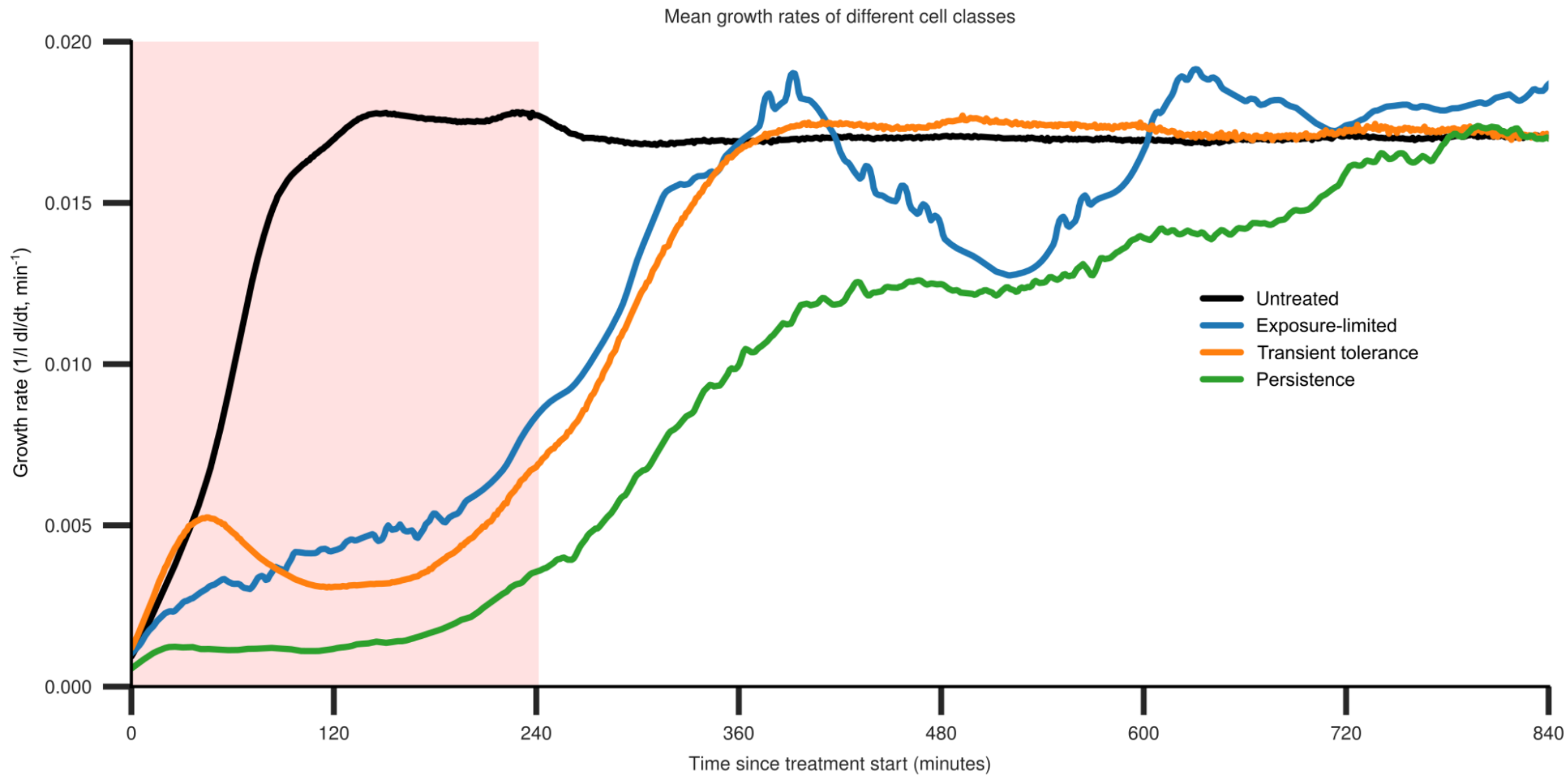

Figure S4: Mean growth rate of different cell classes. Black line indicates untreated cells resuscitated in the absence of an antibiotic. The transient tolerance class starts to follow the same trend as untreated cells or susceptible class, but then slows down growth during treatment and eventually reaches the maximal growth rate after the removal of treatment. Persister cells maintain slow growth throughout treatment and slowly catch up (depending on the distribution of lag time). Exposure-limited survivors are also caused by distributed lag time of the susceptible population and their mean growth rate increases slowly, relative to the susceptible population. Resuscitation was in the presence of no antibiotic (untreated) or 25 or 50 µg/mL ampicillin for 4 hours (red shaded region).

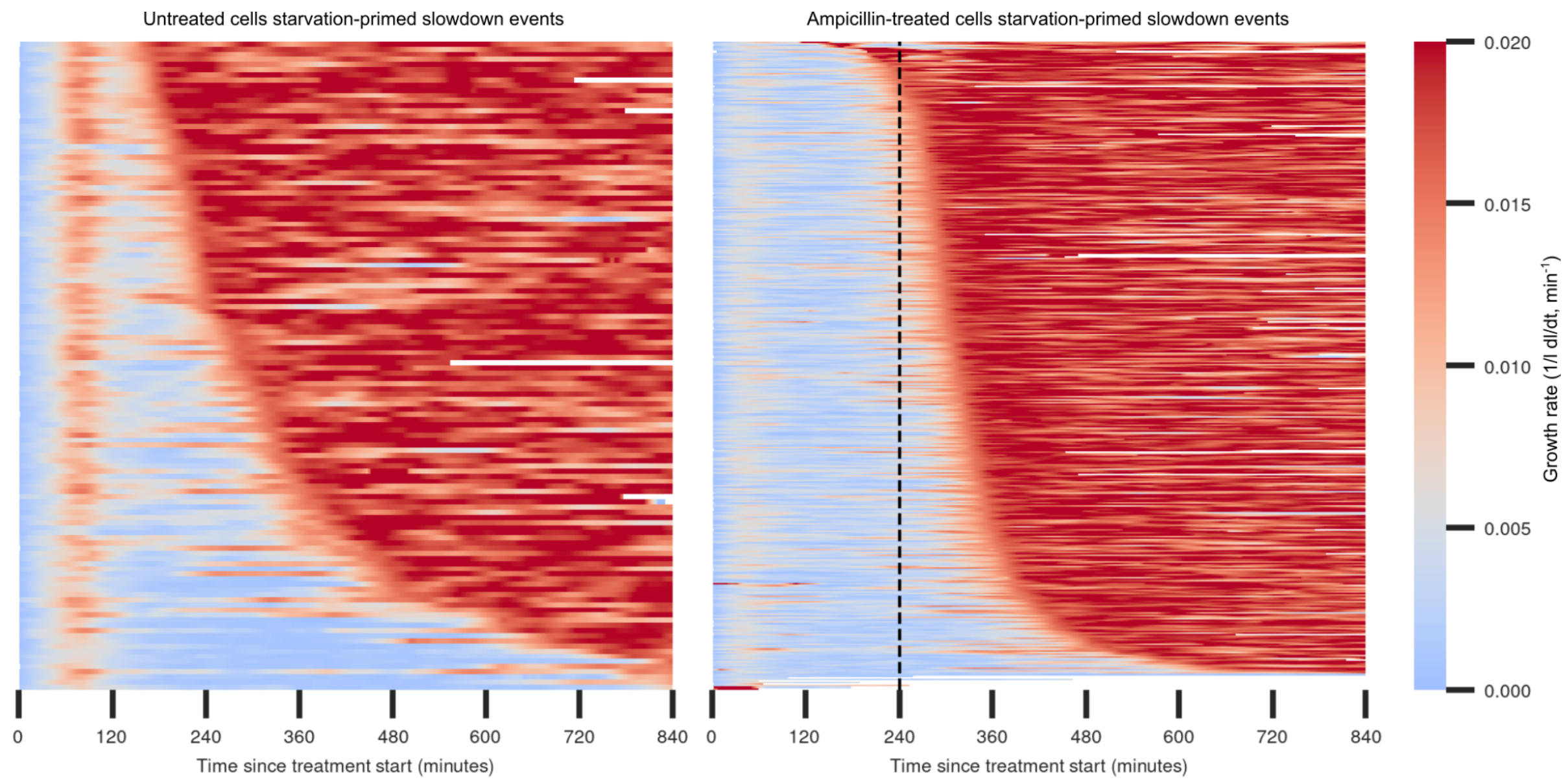

Figure S5: Transient growth slowdown events are present and heterogeneous in the untreated population (left). Heat maps of growth rates of slowdown events in untreated cells compared to the transiently tolerant subpopulation during and after treatment (right). The vertical dashed black line indicates the end of 4 hours of treatment with 25 or 50 µg/mL ampicillin for the transiently tolerant subpopulation.

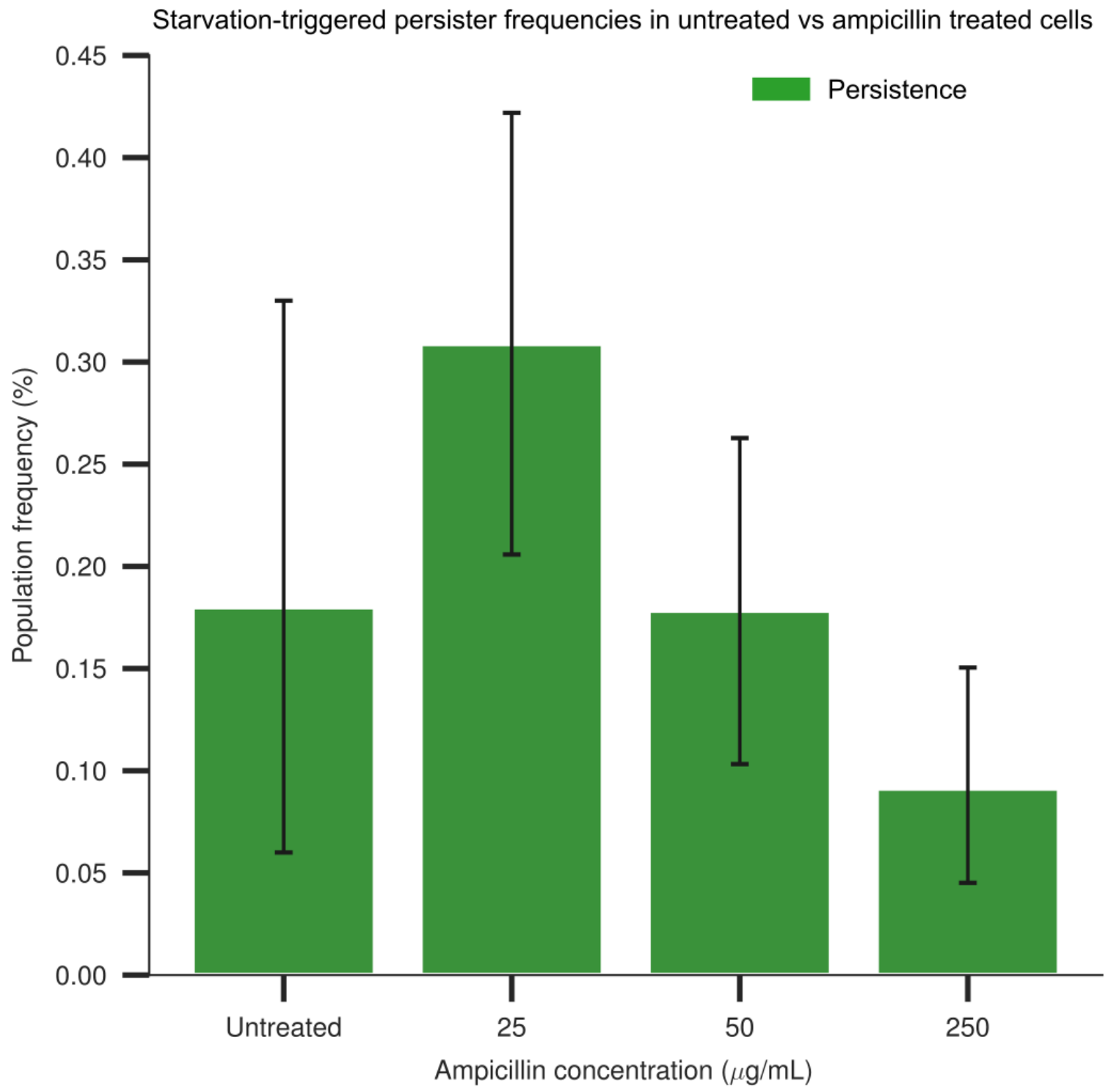

Figure S6: Starvation-triggered persister survivor frequencies of an untreated population of 24 hours old *E. coli*, compared to populations treated with varying concentrations of ampicillin for 4 hours. The initial frequency of starvation-triggered persisters in the population appears to be independent of antibiotic treatment, and hence instead depends on factors such as culture age. Error bars indicate the upper and lower 95% confidence intervals from bootstrapping with $n = 10{,}000$.

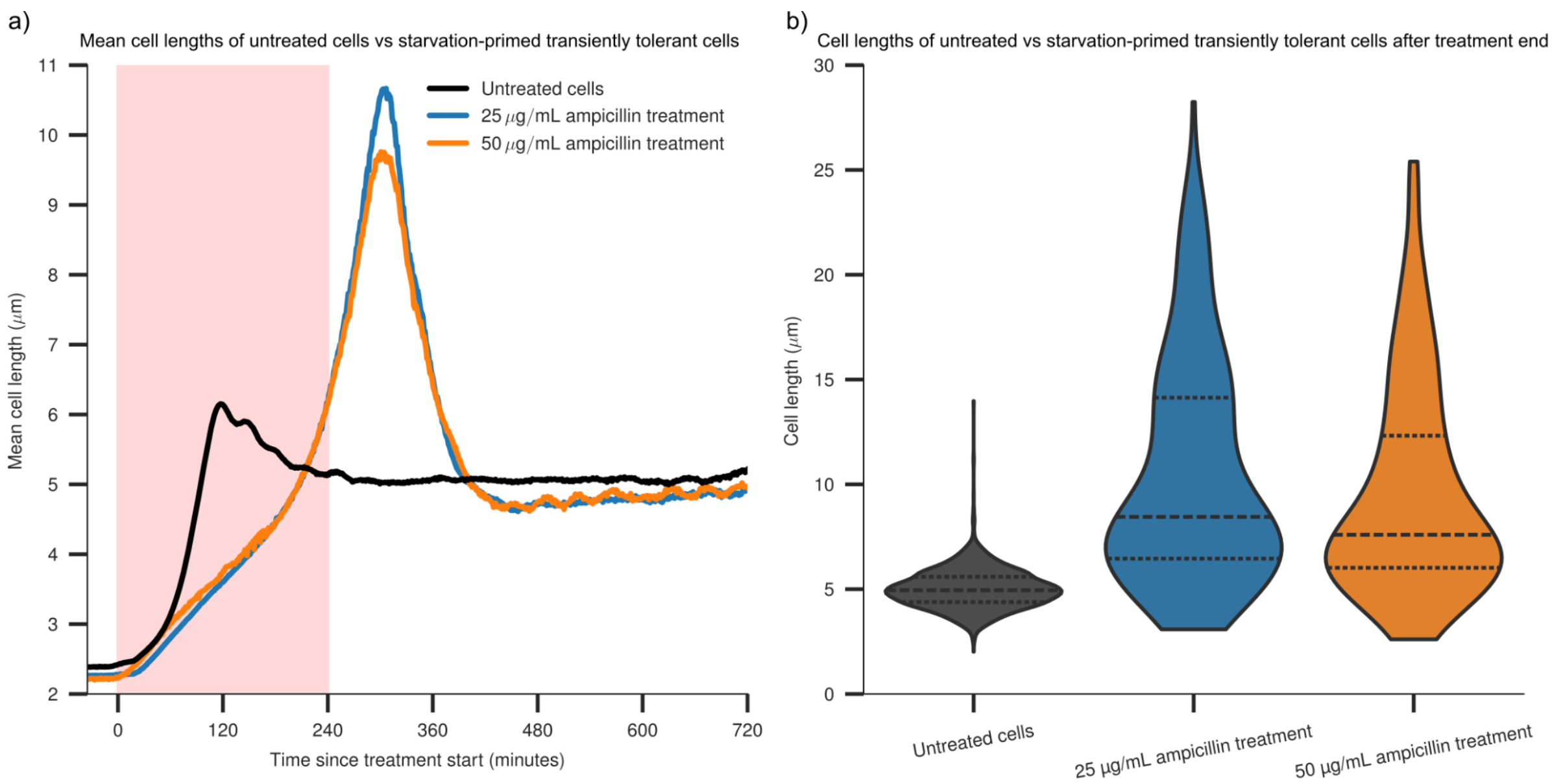

Figure S7: Transiently tolerant cells frequently filament to form long cells that can divide to a large number of progeny following treatment end. a) The mean value of single-cell length traces of a culture of 24 hour old *E. coli* resuscitated in the presence of different treatment conditions. The red shaded region indicates antibiotic treatment. **b)** Violin plots showing the distribution of single-cell lengths 60 minutes after the end of antibiotic treatment (300 minutes after treatment start).

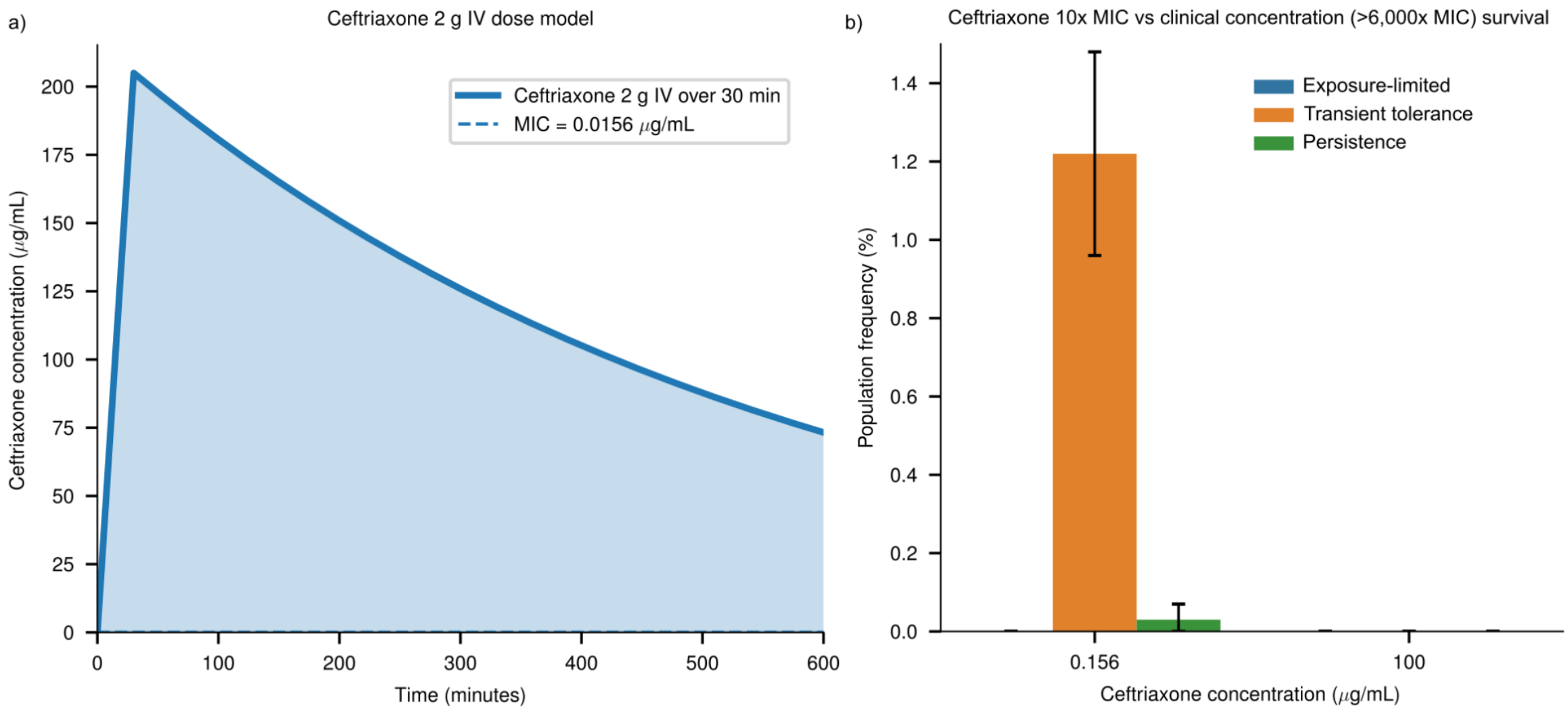

Figure S8: Ceftriaxone clinical pharmacokinetics model and survival at different concentrations. **a)** Pharmacokinetics model of a typical 2 g dose of ceftriaxone administered by IV over 30 minutes. Based on PK parameters from[2,3]. **b)** Survival frequencies of 24 hour old cultures of *E. coli* treated with either 10× MIC (0.156 μg/mL) or the more clinically relevant >6,000× MIC (100 μg/mL) of ceftriaxone for 4 hours. Error bars indicate the upper and lower 95% confidence intervals from bootstrapping with $n = 10{,}000$.

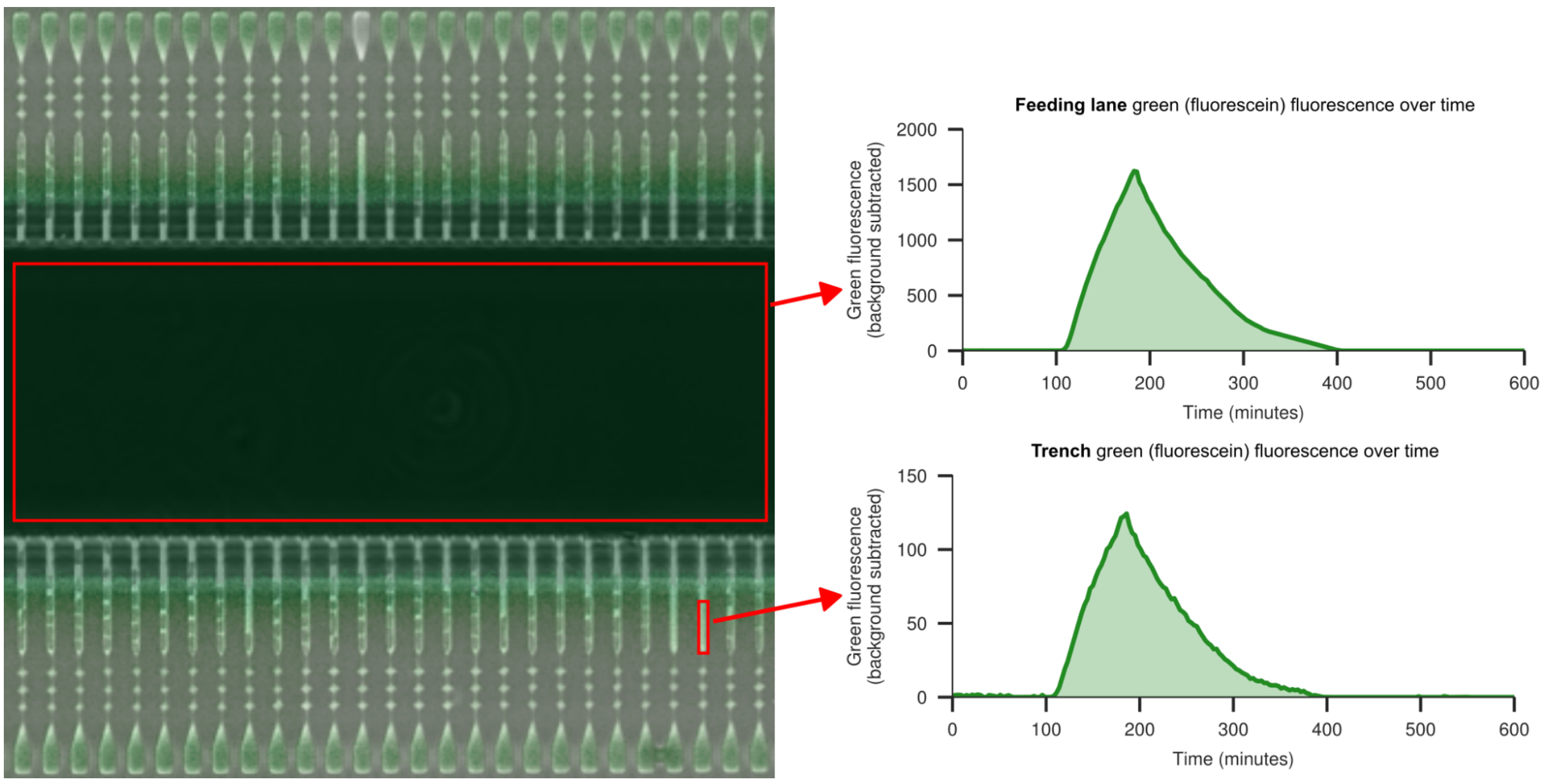

Figure S9: Fluorescein dynamics are consistent between the feeding lane and individual trenches. Overlayed image of the green fluorescence channel and the phase contrast channel of a section of the MMX device 180 minutes into the amoxicillin pharmacokinetics experiment in Figure ??c,d. Plots show dynamics of green fluorescence (due to flowed fluorescein) of the highlighted feeding lane and individual trench sections from 0 to 600 minutes.

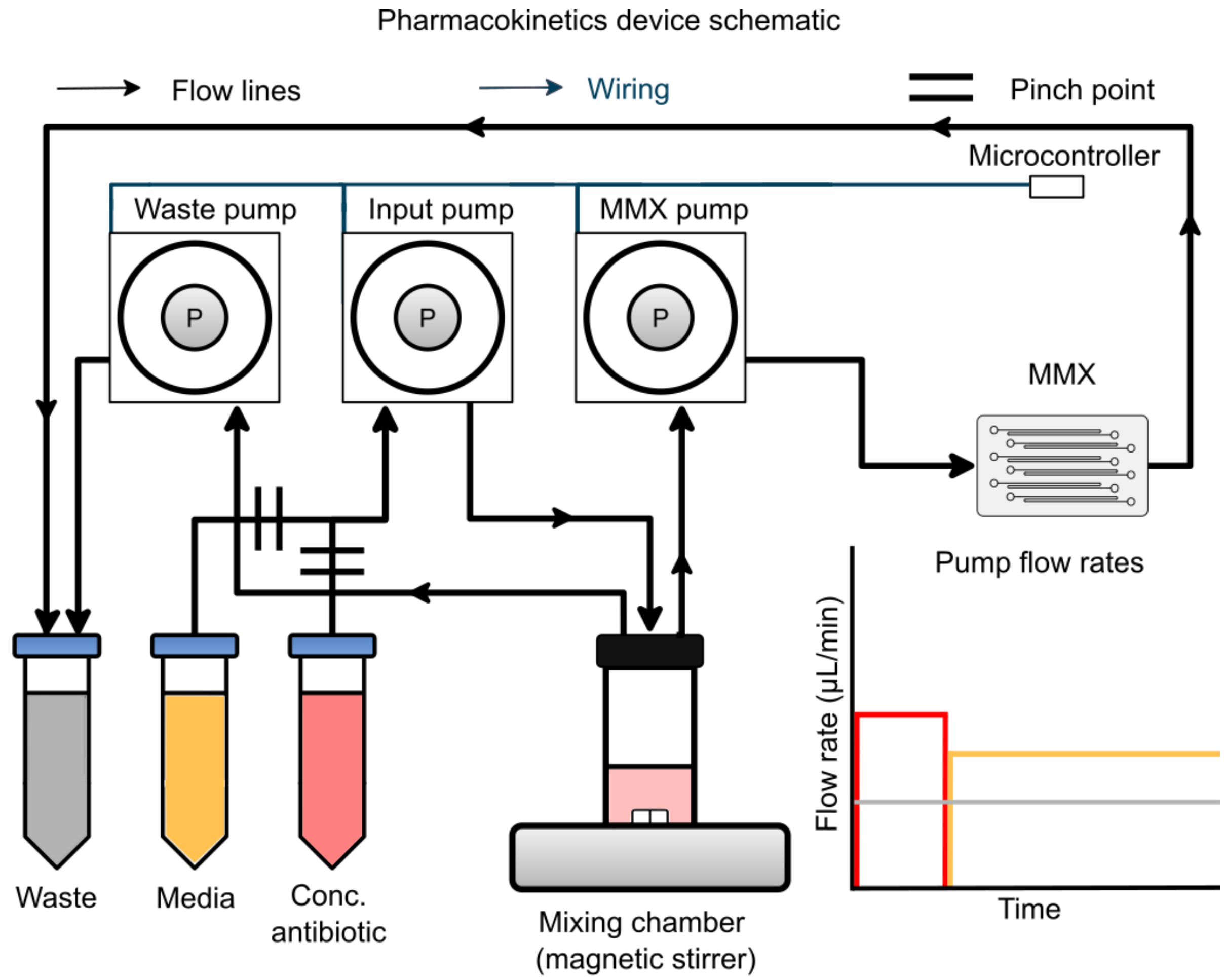

Figure S10: Schematic of the antibiotic pharmacokinetics system. Schematic depicting the multi-pump system for mimicking clinical antibiotic pharmacokinetics within the MMX device. Black lines indicate the direction of liquid flow from a tube containing concentrated antibiotic or from a tube containing fresh supplemented M9 media into a mixing chamber on a magnetic stirrer, which then flows into a MMX device loaded with cells and out to waste. Flow from either the concentrated antibiotic tube or fresh media tubes is controlled by an electronic pinch valve that swaps liquid flow between either concentrated antibiotic or fresh media being added to the mixing chamber.

Supplementary Tables

Table S1: Minimum inhibitory concentration (MIC) of the antibiotics used in this study. *E. coli* MG1655 CGSC 6300 ("SB1") were treated with prepared antibiotic stocks by the broth microdilution method performed as described in the methods section.

Antibiotic	MIC (μg/mL)
Cefalexin	8
Ceftriaxone	0.015625
Amoxicillin	4
Ampicillin	16

Supplementary Videos

Video 1. Time-lapse imaging over a 30-minute period comparing loading dynamics in a MMX-design trench design (right), a MMX-design trench design with a smaller top chamber (middle), and a conventional mother machine trench (left). The MMX enables rapid loading, which is facilitated by evaporatively driven flow, substantially improving efficiency and throughput relative to the conventional, diffusion-based loading approach.

Video 2. Trench images, segmented masks, and corresponding length traces of examples of the different cell classes in response to antibiotic treatment. Red shaded region indicates 4 hours of treatment with 50 μg/mL ampicillin treatment. Top: Susceptible cells rapidly increase in size and subsequently lyse due to the antibiotic. Middle: Transiently tolerant cells initially start to increase in size, then experience a transient growth slowdown that facilitates their survival during antibiotic treatment. These cells often filament to form long cells and subsequently resolve into multiple cells following treatment end. Bottom: Starvation-triggered persisters maintain a slow increase in size from the start of treatment, facilitating their survival during antibiotic treatment, until later rapidly increasing in size.

Supplementary Notes on Methods and Models

Supplementary Note 1: Defining Tolerance, Heterotolerance, Persistence, Transient Tolerance, and Exposure-Limited Survivors

Antibiotic tolerance describes the ability of bacterial cells to survive transient antibiotic exposure without a change in the minimum inhibitory concentration (MIC)[4]. Unlike resistance, tolerance does not alter drug susceptibility at steady state but prolongs survival during treatment. Tolerance can arise through:

- Genetic mechanisms, where mutations alter physiological response to antibiotics.
- Physiological mechanisms, where survival reflects the growth state of cells.

Population-wide nongenetic tolerance:

Beyond genetic tolerance related to specific mutations, a reversible, non-genetic reduction in antibiotic susceptibility can be observed at the bulk-population level due to physiological factors. For example, when environmental conditions suppress essential cellular processes across the entire population (e.g., nutrient deprivation, dormancy, hypoxia), most cells can become tolerant to antibiotics. Recent work[5] has demonstrated that slow or arrested growth at the population level can generate widespread tolerance without invoking phenotypic heterogeneity.

In this scenario:

- The majority of cells are non-growing or slow-growing or activated a specific stress-response, such as stringent response,
- Survival reflects a collective transition where extrinsic stressors, such as nutrient depletion or hypoxia, reprogram the population's physiological state.

Heterotolerance:

We use the term heterotolerance to describe heterogeneous susceptibility within a population arising from phenotypic heterogeneity among cells growing under otherwise permissive conditions.

In heterotolerance:

- The environment supports growth for most cells.
- A subpopulation adopts a physiological state that confers transient survival during antibiotic exposure.

This category includes persisters and the transient tolerance phenotype described here.

Starvation-triggered persistence:

Persisters are a subpopulation of cells that enter a prolonged non-growing or extremely slow-growing state prior to antibiotic exposure. Persisters are typically associated with long lag times following starvation or stress. Starvation-**triggered** persisters refer to the subpopulation of cells that demonstrate extended lag and low growth rates from the start of resuscitation, and delayed regrowth after antibiotic removal[4].

Starvation-primed transient tolerance:

We define **transient tolerance** as a distinct heterotolerant fate manifested during resuscitation following starvation. **Starvation-primed** transient-tolerant cells refer to the subpopulation of cells that initially demonstrate growth rates comparable to the susceptible (or untreated) population at the start of resuscitation, but subsequently experience a transient growth slowdown that facilitates survival during β-lactam antibiotic treatment. Notably, these transient growth slowdown events occur even in the absence of treatment, and their prevalence is scaled by starvation duration. Unlike persisters, transiently tolerant cells:

- Are not deeply dormant.
- Do not exhibit prolonged lag.
- Rapidly rebound after treatment.

Exposure-limited survivors:

Outside of heterotolerance, some cells within a susceptible population are able to survive antibiotic treatment due to limited exposure duration, despite these cells exhibiting the default susceptible phenotype. These exposure-limited survivors arise from natural variability in lag time within the susceptible population: a subset of cells exit lag more slowly and therefore do not reach the growth-dependent threshold required for antibiotic-mediated killing before treatment ends, particularly for short duration antibiotic treatments.

Supplementary Note 2: Hi-DFA analysis pipeline

Microscopy images were saved as nd2 files. The image data and metadata in the nd2 files were extracted, and the images registered using PyStackReg[6] to eliminate stage drift. The trenches within each image were then identified from spikes in the mean x and y intensity profiles of the phase contrast channel, and extracted to a Zarr array[7] using a modified version of PyMMM[8].

The Zarr array of individual trench images was then segmented with Omnipose[9], using a model trained initially on appropriately optimised synthetic trench images generated by SyMBac[10] and subsequently fine-tuned by retraining on manually curated data from the previous iteration of the model output. Prior to segmentation, trench images were upscaled 2× in each spatial dimension using bicubic interpolation, yielding images of typical size 280×48 pixels from the native 140×24 pixels. This upscaling reduces the effect of pixelation for the small cell sizes typical of stationary-phase and early-resuscitation cells. The segmentation model was trained on equivalently upscaled images. Omnipose was trained for 4000 epochs, using default settings from the paper[9]. Segmentation masks were also saved as Zarr arrays.

Following image segmentation, small segmentation artefacts were removed by applying a minimum size threshold for masks, just below the size of the smallest real cell masks. Additional filters were applied for a minimum solidity threshold (which quantifies how convex masks are, calculated by regionprops from scikit-image[11]) and minimum distance from the edges of the image (to exclude segmentation artefacts outside of the trench).

The filtered segmentation masks were used to extract quantitative image features of the mother cell, using the mask at the top of the corresponding trench images, using regionprops. Extracted features typically included area, length, width, image intensity, solidity. The extracted data from regionprops was stored in a pandas dataframe, saved as a pickle file.

The data from the pickle file was subsequently used for dynamic fate analysis. Details on the time and treatment condition for each trench were added as metadata to the corresponding cell IDs of the dataframe. To identify trenches that contained at least one cell at the start of the experiment, we used a mask threshold, such that the trench could not lack a mask in more than 10% of the first 20 frames. A similar threshold was used to identify trenches that contained a cell at the end of the experiment, to minimise including segmentation artefacts.

The time-series of the measured length for each cell (which is the primary value used for subsequent analysis) then went through several cleanup steps. These cleanup steps included setting minimum length thresholds (to remove impossibly small cells, i.e. segmentation artefacts) and identifying positive and negative spikes where the length significantly increased/decreased then quickly reverted.

After these initial cleanup steps, division events were identified using SciPy[12] `find_peaks` function on the negative first derivative of the cleaned length data against time. A minimum prominence value was applied for identifying sufficiently large peaks (whilst excluding as many artefact peaks as possible), a minimum time between division events and minimum and maximum division event sizes were also applied to further minimise incorrectly identified division events.

To minimise segmentation noise in the length traces affecting calculated growth rates, the cleaned length trace data was smoothed using LOWESS smoothing (using a window size fraction set to a fixed number of timepoints, typically those comprising the surrounding ~20 minutes of timepoints). Cell length drops owing to division events were temporarily “removed” to bring the divided cell lengths up to their pre-division lengths, then LOWESS smoothing was applied, before “adding back” the division events to the smoothed length traces.

Similar data cleanup parameters were used for all experiments, with the exception of the supra-MIC experiment which had 70 second frame intervals, instead of the typical 3 minute frame intervals, requiring appropriate modifications.

Growth rate was then calculated as the first derivative of the smoothed cell length traces and similar LOWESS smoothing was also applied to the growth-rate timeseries. The second derivative of the length trace was then calculated by taking the derivative of the growth rate trace, and light LOWESS smoothing applied. Growth slowdown events were identified from the second derivative of cell length timeseries. A slowdown was defined as a sustained drop, when the derivative remained below 0 (i.e. growth rate is slowing down) for above a threshold amount (defined by the integrated area above the curve). The growth slowdown was identified as terminated when the second derivative subsequently remained above 0 for a threshold amount (defined by the area below the curve exceeding a threshold amount relative to the identified accumulated negative area above the curve).

Cell survival was defined by the occurrence of a threshold number of division events following antibiotic treatment, typically assessed during the final 6 hours time window of the experiment. This threshold number of division events was chosen to be fairly generous, to also allow identification of slow growing cells or occasional missed division events during poorly segmented stretches. A filter for maximum % of acceptable NaN timepoints was also applied to minimise division event artefacts being included.

Following identification of cells surviving antibiotic treatment, to minimise the artefacts included in subsequent analysis, the trench images, corresponding masks, and corresponding growth traces were typically opened for each “survivor” in Napari[13], and Napari hotkeys were used to quickly classify ‘survivors’ as real or artefacts; this includes discarding cells in the rare case where the mother cell at the top of the trench dies, and one of the cells below the mother cell replaces it. During this manual data cleanup step, we typically also visually identified and classified the slow-growing starvation-triggered persisters, using a different hotkey. We note that based on the manually classified starvation-triggered persisters, an automatic classification criteria for this class of a mean growth rate below 0.0021 min^{-1} (~10% of the maximum growth rate) for the first 120 minutes after introducing fresh media, and also for the first 60 minutes after introduction of fresh media (to exclude transient tolerant survivors), matches the manual classification quite well.

Survivors that were not classified as starvation-triggered persisters were then classified as either transiently tolerant survivors or exposure-limited survivors, based on the presence/absence of a significant growth slowdown event prior to the end of antibiotic treatment. Transiently tolerant survival events were identified by the presence of a slowdown event during antibiotic treatment that increased the integrated lost time of the cell from growth slowdown (using a similar definition to[1]) by at least 10 minutes.

After identifying cell classes, plots of cell length traces against time were created to visually confirm that everything seemed correct. Occasionally, a small number of “exposure-limited” traces that visibly showed growth slowdown, but were not classified as having significant slowdown events (usually due to segmentation errors), were manually classified into the transiently tolerant class.

After confirming cell classes, confidence intervals were calculated by bootstrapping with $n = 10{,}000$. These bootstrapped mean values and 95% confidence intervals were then used for subsequent analysis between different treatment conditions. Calculated growth rates were used for comparison between cell classes, or treatment conditions. To ensure that mean growth rate traces/heatmaps were minimally

affected by artefacts, a small number of growth rate traces that clearly didn't fit the raw data well (usually due to the raw data being messy, owing to occasional messy segmentation), were manually excluded from these analyses.

The Hi-DFA pipeline is available at `https://github.com/kieranrabbott/Hi-DFA`.

Supplementary Note 3: Antibiotic pharmacokinetics system design and assembly

Hardware Configuration of the PK simulator. To simulate clinically relevant pharmacokinetic (PK) profiles in the microfluidic experiments, we developed a programmable flow-based system comprising three peristaltic pumps (Longer T100 & WX10 OEM), a mixing chamber, and a set of solenoid pinch-valve-controlled inlets for precise modulation of antibiotic concentration over time (schematic of the setup shown in Figure S10). The configuration was designed to deliver continuously varying antibiotic concentrations to the microfluidic device (MMX) while maintaining constant flow and volume within the system.

Two pumps were configured as outlet pumps, one directed flow from the mixing chamber through the MMX device, and the second removed excess liquid to waste, thereby maintaining stable pressure and flow conditions. A third inlet pump supplied media to the mixing chamber, which received input from either a fresh (antibiotic-free) or a concentrated (antibiotic-containing) medium source. These two input lines were joined via a Y-junction upstream of the mixing chamber. Flow selection between the fresh and concentrated inputs was controlled by a solenoid pinch valve (Cole-Parmer VapLock), which switched the inlet state according to a programmed time sequence to reproduce desired concentration-time profiles.

The mixing chamber consisted of a 20 mL borosilicate glass vial fitted with a custom cap containing three 0.75 mm-radius ports for inlet, outlet, and waste connections. A small magnetic stir bar placed inside the vial ensured rapid and complete mixing of the incoming streams when coupled with an external magnetic stirrer. The chamber thus acted as a continuously stirred reservoir where the antibiotic concentration evolved dynamically based on the inlet flow composition and dilution rate, reproducing exponential decay or complex clinical PK profiles as required.

Fluidic connections were made using 3/32 inch ID tubing. The outlet tubing was positioned below a 5 mL liquid line inside the chamber to ensure continuous withdrawal of liquid and to maintain a constant working volume. Flow rates for the inlet and outlet pumps were calibrated to match, maintaining a steady-state flow through the device. The concentration trajectory within the mixing chamber was verified experimentally using fluorescence dyes under identical flow conditions.

***in vitro* PK Simulator Logic.** The PK setup was designed to be as minimal and reproducible as possible (Figure **??**a), based on first-order pharmacokinetics to model both the concentration build-up (step-up to $C_{\max}$) and dilution (step-down) or the half-life. Pump speeds in each regime are controlled by an ESP32 microcontroller that encodes the calculated flow rates and their previously chosen time constants.

For example, for amoxicillin requiring approximately ~80 minutes to achieve $C_{\max}$, the relationship between concentration and volume is given by:

$$C_1 V_1 = C_2 V_2, \quad V_1 = \frac{C_2 V_2}{C_1} \tag{S1}$$

Substituting C_2 (desired conc.) 2.5 mmol, V_2 (initial volume) 5 mL, C_1 (initial conc.):

$$V_1 = \frac{1 \text{ mM} \times 5 \text{ mL}}{2.5 \text{ mM}} = 2 \text{ mL} \tag{S2}$$

Flowrate Determination. The flowrate required to replace this calculated volume and achieve the desired concentration within the targeted time (t) is given by:

$$Q = \frac{V}{t} = \frac{2 \text{ mL}}{80 \text{ min}} = 0.025 \text{ mL min}^{-1} = 25.0 \text{ µL min}^{-1} \tag{S3}$$

Where Q is the flowrate, maintaining constant volume requires:

$$(Q'_{\text{MMX}} + Q'_{\text{Waste}}) - Q'' = 0 \text{ µL min}^{-1} \tag{S4}$$

where Q' is the output media and Q'' is the input media (to the mixing chamber); Q'_{MMX} was fixed at 10 µL min^{-1}.

Half-life ($t_{1/2}$) flow profile. For calibration and validation of the pharmacokinetic (PK) setup, fluorescein was used as a non-reactive fluorescent tracer to quantify the generated concentration-time profiles of the antibiotics. The fluorescein intensity in the MMX device was continuously measured by fluorescence imaging to confirm that the system reproduced the intended temporal decay and steady-state characteristics under each programmed condition. In the half-life simulation phase, the objective was to achieve a dilution time constant equivalent to the reported pharmacokinetic half-life ($t_{1/2}$) of the tested antibiotic. Here, $t_{1/2}$ was defined as the time required to replace half of the total chamber volume (V) with fresh medium. Using the flow-volume relationship the system's embedded ESP32 microcontroller dynamically adjusted the pulse-width modulation (PWM) speed of each peristaltic pump to reproduce a first-order exponential decay of concentration over time. This enabled precise control of the antibiotic exposure profile, ensuring that the concentration delivered to the microfluidic device closely matched clinically relevant elimination kinetics. The ESP32 adjusted each pump's PWM speed according to the required first-order decay profile.

Flowrate Calibration. Before each experiment, a series of flowrate calibration runs are completed, in which a Sensirion SLF3S-0600F flowrate monitor is connected to each fluidic line's flow output and is monitored mapping PWM (Pulse Width Modulation) to µL min^{-1}. The flowrate of which is recorded and PWM is tuned toward the desired value with a grid-search algorithm. Each pump is done accordingly, until the waste and fresh pumps are within ± 1 µL min^{-1} and the MMX pump is within ± 0.3–0.5 µL min^{-1}. Sterilization is then conducted on the system as outlined in the methodology section and spent media is flowed through the system and the mixing chamber is filled with fresh M9 media.

Supplementary Note 4: Mathematical Model of Transient Tolerance

1 General Model Framework

1.1 States and transitions

We consider a population of N_0 cells at $t = 0$, the onset of antibiotic treatment and growth media replenishment, which can trigger exit from dormancy, at constant concentration C for duration τ. Each cell can occupy one of three states: dormant (D, not yet exited stationary phase), susceptible (S, actively growing and drug-susceptible), or transiently tolerant (T, actively growing with reduced drug susceptibility). Cells that die from either S or T enter an absorbing dead state. The correspondence between these model states and the experimentally observed post-treatment survivor classes is summarised in Table S2.

Table S2: Mapping between experimental survivor classes and model states. The three post-exposure survivor classes—persisters ($P \subset D$), exposure-limited survivors ($X \subset S$), and transiently tolerant survivors (T)—emerge naturally from the three model states D, S, and T, respectively (Figure 7c).

Experimental term	Definition	Model state
Lag-based survivor (incl. starvation-triggered persister, $P \subset D$)	Does not exit dormancy within $[0, \tau]$ ($L > \tau$); the extreme tail corresponds to classical starvation-triggered persisters	D
Exposure-limited survivor ($X \subset S$)	Wakes under drug, remains in S without switching, survives by timing (short τ or low C)	S
Transiently tolerant survivor (T)	Wakes, transiently reduces susceptibility, survives with reduced but non-zero death rate	T

The population dynamics during the treatment phase (we consider transitions only during antibiotic exposure, not during post-treatment regrowth; see §3 for regrowth dynamics) are governed by

$$\frac{dD}{dt} = -h_L(t; a)\, D, \tag{S5}$$

$$\frac{dS}{dt} = h_L(t; a)\, D - h_S(C)\, S - r_{S\to T}(C, a)\, S, \tag{S6}$$

$$\frac{dT}{dt} = r_{S\to T}(C, a)\, S - h_T(C)\, T, \tag{S7}$$

with initial conditions $D(0) = N_0$, $S(0) = T(0) = 0$, and where:

- $h_L(t; a)$: instantaneous hazard of exiting dormancy at time t, given culture age a;
- $h_S(C)$: death rate of susceptible cells;
- $h_T(C)$: death rate of transiently tolerant cells, with $0 < h_T(C) < h_S(C)$ for all $C > 0$;
- $r_{S\to T}(C, a)$: rate of switching from S to T.

We impose no back-transition $T \to S$, as we only consider transitions during the treatment phase, and this is consistent with the observation that cells entering the transiently tolerant state do not revert to full susceptibility during the treatment window, but rather die at their reduced growth rate as compared to fully susceptible cells.

1.2 Lag-time distribution

Individual lag times L follow a two-component mixture of an Erlang distribution (weight w, shape k, rate $\lambda(a)$) and a slow exponential (weight $1-w$, rate λ_s):

$$f_L(t;a) = w \cdot f_{\text{Erlang}}(t;\, k, \lambda(a)) + (1-w) \cdot \lambda_s\, e^{-\lambda_s t}, \tag{S8}$$

where the Erlang component has PDF, CDF, and survival function

$$f_{\text{Erlang}}(t;a) = \frac{\lambda(a)^k\, t^{k-1}\, e^{-\lambda(a)t}}{(k-1)!}, \qquad t \geq 0, \tag{S9}$$

$$F_{\text{Erlang}}(t;a) = 1 - e^{-\lambda(a)t} \sum_{m=0}^{k-1} \frac{(\lambda(a)t)^m}{m!}, \tag{S10}$$

$$S_{\text{Erlang}}(t;a) = e^{-\lambda(a)t} \sum_{m=0}^{k-1} \frac{(\lambda(a)t)^m}{m!}. \tag{S11}$$

The overall survival function for the hybrid mixture is

$$S_L(\tau;a) = w \cdot S_{\text{Erlang}}(\tau;a) + (1-w) \cdot e^{-\lambda_s \tau}. \tag{S12}$$

The mean lag time of the Erlang component is $\mu_{\text{lag}}(a) = k/\lambda(a)$, modelled as a linear function of culture age:

$$\mu_{\text{lag}}(a) = \mu_0 + \Delta\mu_{24+}\left(\frac{a}{24}\right), \tag{S13}$$

where μ_0 is the baseline mean lag for a fresh culture and $\Delta\mu_{24+}$ is the additional mean lag accumulated per 24 h of stationary phase. This gives $\lambda(a) = k/\mu_{\text{lag}}(a)$.

The overall mean lag time under the mixture is $\mathbb{E}[L] = w \cdot \mu_{\text{lag}}(a) + (1-w)/\lambda_s$, and we define the *effective lag rate*

$$\lambda_{\text{eff}}(a) = \frac{1}{\mathbb{E}[L]} = \frac{1}{w \cdot \mu_{\text{lag}}(a) + (1-w)/\lambda_s}, \tag{S14}$$

which replaces the bare Erlang rate $\lambda(a)$ in all constraint penalties (§5). Unlike the Erlang rate $\lambda(a)$, the slow-exit rate λ_s is modelled as age-independent: the deeply dormant subpopulation captured by this component is assumed to have entered a state whose exit kinetics are insensitive to the additional starvation stress accumulated over the culture ages tested. When $w \approx 0$, the Erlang component is negligible and $\lambda_{\text{eff}} \approx \lambda_s$; when $w = 1$, $\lambda_{\text{eff}} = \lambda(a) = k/\mu_{\text{lag}}(a)$.

The Erlang shape parameter k (selected by grid search over $k = 2, \ldots, 14$) controls the regularity of wake times in the main component: larger k produces a sharper distribution around the mean (coefficient of variation $= 1/\sqrt{k}$). The slow-exponential component (rate λ_s) captures a subpopulation of cells with extended lag times that are not well described by the Erlang tail; this heavy-tailed subpopulation corresponds to the slow phase of classical biphasic killing and includes cells that would conventionally be classified as starvation-triggered persisters.

1.3 Hazard functions

The concentration-dependent transition rates are expressed in terms of generic antibiotic pressure functions $\varphi(C)$ that are monotonically increasing with $\varphi(0) = 0$:

$$h_S(C) = \alpha_S\, \varphi_S(C), \tag{S15}$$

$$h_T(C) = \alpha_T\, \varphi_T(C), \tag{S16}$$

$$r_{S\to T}(C, a) = m(a)\big[r_0 + \alpha_{ST}\, \varphi_{ST}(C)\big], \tag{S17}$$

where $\alpha_S, \alpha_T, \alpha_{ST}$ are rate constants and $r_0 > 0$ is a baseline switching rate that accounts for transient growth-slowdown events observed even in untreated resuscitating cells (Figure S5). The stress-history function $m(a) = a/(a + a_{50})$ with $m \in [0, 1)$ increases monotonically with culture age, capturing the observation that older cultures produce more transiently tolerant survivors. We impose $\alpha_S > \alpha_T > 0$ so that $h_S(C) > h_T(C)$ (for our choices of $h_S(C), h_T(C)$ described below) for all $C > 0$ when $\varphi_S = \varphi_T$.

2 Analytical Survivor Fractions

2.1 Convolution structure

A key property of the system (S5)–(S7) is that h_S, h_T, and $r_{S\to T}$ are constant during the treatment window (constant C and a). Only the dormancy-exit hazard $h_L(t; a)$ varies with time, so the S/T subsystem is a linear, time-invariant system driven by the time-varying inflow from D.

Solving (S5) gives $D(t) = N_0\, S_L(t; a)$, so the inflow rate into S at time t is $N_0 f_L(t; a)$. The surviving populations in S and T at time t are therefore convolutions:

$$S(t) = N_0 \int_0^t P_S(t - \ell)\, f_L(\ell; a)\, d\ell, \tag{S18}$$

$$T(t) = N_0 \int_0^t P_T(t - \ell)\, f_L(\ell; a)\, d\ell, \tag{S19}$$

where $P_S(\Delta)$ and $P_T(\Delta)$ are single-cell survival kernels for a cell exposed for duration Δ, derived below.

2.2 Derivation of the survival kernels

Consider a cell entering state S at time ℓ, subsequently exposed to antibiotic for duration $\Delta = \tau - \ell$. Define the total exit rate from S:

$$H = h_S(C) + r_{S\to T}(C, a). \tag{S20}$$

Kernel $P_S(\Delta)$: surviving in S. The probability of remaining in S (neither dying nor switching) satisfies

$$\frac{dP_S}{d\Delta} = -H\, P_S, \qquad P_S(0) = 1, \tag{S21}$$

giving

$$\boxed{P_S(\Delta) = e^{-H\Delta}.} \tag{S22}$$

Kernel $P_T(\Delta)$: switching to T and surviving. A cell becomes a T-survivor by first switching $S \to T$ at some time $t \in [0, \Delta]$, then surviving in T for the remaining $\Delta - t$. The probability density for the switch occurring at time t, given that the cell is still in S at time t, is

$$f_{\text{switch}}(t) = r_{S\to T}\, e^{-Ht}, \qquad 0 \le t \le \Delta, \tag{S23}$$

since e^{-Ht} is the probability of no event (neither death nor switch) in S up to t, and $r_{S\to T}$ is the instantaneous switching rate. Conditional on switching at t, survival in T for the remaining $\Delta - t$ is $e^{-h_T(\Delta - t)}$. Integrating over all possible switch times:

$$\begin{aligned} P_T(\Delta) &= \int_0^\Delta r_{S\to T}\, e^{-Ht}\, e^{-h_T(\Delta-t)}\, dt \\ &= r_{S\to T}\, e^{-h_T\Delta} \int_0^\Delta e^{-(H-h_T)t}\, dt. \end{aligned} \tag{S24}$$

Evaluating the integral for the generic case $H \neq h_T$ and the degenerate case $H = h_T$:

$$\boxed{P_T(\Delta) = \begin{cases} \dfrac{r_{S\to T}}{H - h_T}\left[e^{-h_T\Delta} - e^{-H\Delta}\right] & \text{if } H \neq h_T, \\ r_{S\to T}\, \Delta\, e^{-h_T\Delta} & \text{if } H = h_T. \end{cases}} \tag{S25}$$

The remaining probability $1 - P_S(\Delta) - P_T(\Delta)$ corresponds to death during exposure.

2.3 Population fractions at end of treatment

At time τ, the population is partitioned into four classes (Figure 7c):

Lag-based survivors ($P \subset D$). Cells that have not yet resuscitated by the end of treatment ($L > \tau$), the extreme tail of which corresponds to classical starvation-triggered persisters:

$$\pi_D(a, \tau) = S_L(\tau; a) = 1 - F_L(\tau; a). \tag{S26}$$

Exposure-limited survivors ($X \subset S$). Cells that resuscitated during treatment ($L \le \tau$), entered S, and survived in S until τ without switching to T. These are not a mechanistically distinct class, but rather cells whose growth trajectory and treatment timing allowed them to escape killing:

$$\pi_S(C, \tau, a) = \int_0^\tau P_S(\tau - \ell)\, f_L(\ell; a)\, d\ell. \tag{S27}$$

Transiently tolerant survivors (T). Cells that resuscitated, switched to T, and survived in T until τ:

$$\pi_T(C, \tau, a) = \int_0^\tau P_T(\tau - \ell)\, f_L(\ell; a)\, d\ell. \tag{S28}$$

Dead. $\pi_{\text{dead}} = 1 - \pi_D - \pi_S - \pi_T$.

Remark. These expressions are unconditional population fractions (over the entire initial population). They can equivalently be derived by writing the conditional survivor fractions among resuscitating cells (those with $L \le \tau$) and multiplying by the resuscitation probability $F_L(\tau; a)$; the prefactors cancel.

2.4 Closed-form evaluation

Define the *discounted wake integral*

$$\phi(\alpha, \tau) \equiv \int_0^\tau e^{-\alpha(\tau-\ell)} \, f_L(\ell; a) \, d\ell, \tag{S29}$$

which has a direct physical interpretation: $\phi(\alpha, \tau)$ is the total surviving mass of cells that woke during $[0, \tau]$ and experienced a constant-rate clearance process with rate α for their remaining exposure time. The survivor fractions then take the compact forms

$$\pi_S = \phi(H, \tau), \qquad \pi_T = \frac{r_{S\to T}}{H - h_T} \big[\phi(h_T, \tau) - \phi(H, \tau)\big]. \tag{S30}$$

For the hybrid lag distribution (S8), ϕ decomposes as a weighted sum:

$$\phi(\alpha, \tau) = w \cdot \phi_{\mathrm{Erl}}(\alpha, \tau) + (1 - w) \cdot \phi_{\mathrm{Exp}}(\alpha, \tau), \tag{S31}$$

where the Erlang component is evaluated via the J_m recurrence:

$$\phi_{\mathrm{Erl}}(\alpha, \tau) = \frac{\lambda^k}{(k-1)!} J_k, \qquad J_1 = \frac{e^{-\lambda\tau} - e^{-\alpha\tau}}{\alpha - \lambda}, \qquad J_m = \frac{\tau^{m-1} e^{-\lambda\tau} - (m-1)\, J_{m-1}}{\alpha - \lambda}, \tag{S32}$$

and the exponential component has the closed form

$$\phi_{\mathrm{Exp}}(\alpha, \tau) = \frac{\lambda_s \big(e^{-\lambda_s\tau} - e^{-\alpha\tau}\big)}{\alpha - \lambda_s}. \tag{S33}$$

When $\alpha \approx \lambda$ or $\alpha \approx \lambda_s$, Taylor expansions are used to avoid catastrophic cancellation (threshold $|\Delta|\tau < 10^{-8}$). In practice, the product $e^{-\alpha\tau} \cdot J_k$ is evaluated by carrying the exponential factor into the recurrence, operating on the numerically stable difference $e^{-\lambda\tau} - e^{-\alpha\tau}$.

3 Post-Treatment Regrowth Dynamics

3.1 Setup

At $t = \tau$ the antibiotic is removed. Let $u = t - \tau \ge 0$ denote the time since drug removal. Both transiently tolerant and exposure-limited survivors have already exited dormancy during treatment; thus, upon drug removal, transiently tolerant cells are assumed to revert immediately to a growing phenotype, so that all awakened survivors resume exponential growth immediately (residual lag $L_{\mathrm{res}} = 0$):

$$G_X(u) = N_0 \, \pi_S \, e^{gu}, \qquad G_T(u) = N_0 \, \pi_T \, e^{gu}. \tag{S34}$$

Lag-based survivors (including persisters, $P \subset D$), by contrast, retain a residual lag of $L_{\text{res}} = L - \tau$: they must first complete the remaining stages of their dormancy exit before contributing to the growing population. This delay is the key asymmetry: transiently tolerant and exposure-limited survivors have a head start in seeding regrowth.

3.2 Residual lag via the linear-chain trick

The Erlang component of the hybrid lag distribution (weight w, shape k) can be represented as the sum of k independent exponential stages, each with rate λ. The exponential component (weight $1 - w$, rate λ_s) contributes a single additional stage. At $u = 0$, the surviving dormant mass $N_0 \pi_D$ is distributed across these stages according to the hybrid survival function (S12).

For the Erlang component, the linear-chain dynamics are:

$$\frac{dD_1}{du} = -\lambda D_1, \qquad D_1(0) = N_0\, w\, e^{-\lambda\tau}, \tag{S35}$$

$$\frac{dD_i}{du} = \lambda D_{i-1} - \lambda D_i, \quad i = 2, \ldots, k, \qquad D_i(0) = N_0\, w\, e^{-\lambda\tau} \frac{(\lambda\tau)^{i-1}}{(i-1)!}, \tag{S36}$$

$$\frac{dG_{P,\text{Erl}}}{du} = g\, G_{P,\text{Erl}} + \lambda\, D_k, \qquad G_{P,\text{Erl}}(0) = 0. \tag{S37}$$

For the exponential component:

$$\frac{dD_s}{du} = -\lambda_s D_s, \qquad D_s(0) = N_0\,(1 - w)\, e^{-\lambda_s\tau}, \tag{S38}$$

$$\frac{dG_{P,\text{Exp}}}{du} = g\, G_{P,\text{Exp}} + \lambda_s\, D_s, \qquad G_{P,\text{Exp}}(0) = 0. \tag{S39}$$

The total growing pool from persisters is $G_P = G_{P,\text{Erl}} + G_{P,\text{Exp}}$. One can verify that the initial masses sum correctly: $\sum_{i=1}^{k} D_i(0) + D_s(0) = N_0\, S_L(\tau; a) = N_0\, \pi_D$.

3.3 Dormant descendant contribution

By variation of parameters:

$$G_P(u) = N_0\, e^{gu} \int_0^u e^{-gs}\, f_L(\tau + s)\, ds. \tag{S40}$$

This integral accounts for the fact that lag-based survivors (including persisters) that complete their residual lag at post-treatment time s contribute to the growing population, but their descendants are discounted by the factor e^{-gs} relative to cells that were already growing from $u = 0$. For the hybrid lag distribution (S8), both the Erlang and exponential components contribute independently to this integral.

3.4 Descendant shares

The total regrowing population is $N(u) = G_X(u) + G_T(u) + G_P(u)$. The fractional contribution of each survivor class is

$$w_X(u) = \frac{G_X(u)}{N(u)}, \qquad w_T(u) = \frac{G_T(u)}{N(u)}, \qquad w_P(u) = \frac{G_P(u)}{N(u)}. \tag{S41}$$

Cancelling the common factor $N_0 e^{gu}$:

$$w_T(u) = \frac{\pi_T}{\pi_S + \pi_T + J_g(u)}, \tag{S42}$$

where

$$J_g(u) = \int_0^u e^{-gs} f_L(\tau + s)\, ds. \tag{S43}$$

Since $J_g(0) = 0$ and $J_g(u)$ increases monotonically to its asymptotic value $J_g(\infty)$, the transiently tolerant share $w_T(u)$ is maximal immediately after drug removal and decreases as lag-based survivors (persisters) progressively complete their residual lag and enter the growing population. The structure of Equation (S42) makes explicit that transiently tolerant-descended cells dominate early regrowth whenever π_T is comparable to or exceeds π_S, because persisters ($P \subset D$) contribute only with a delay penalised by their residual lag $L_{\text{res}} = L - \tau$.

For the hybrid lag distribution, $J_g(u)$ decomposes into Erlang and exponential contributions:

$$J_g(u) = w \cdot J_{g,\text{Erl}}(u) + (1 - w) \cdot J_{g,\text{Exp}}(u), \tag{S44}$$

where the Erlang component is evaluated using the regularised incomplete gamma function with $\alpha = \lambda + g$:

$$J_{g,\text{Erl}}(u) = \left(\frac{\lambda}{\alpha}\right)^k e^{g\tau} \left[P(k, \alpha(\tau + u)) - P(k, \alpha\tau)\right], \tag{S45}$$

where $P(k, x) = \gamma_{\text{reg}}(k, x)$ is the regularised lower incomplete gamma function, and the exponential component has the closed form

$$J_{g,\text{Exp}}(u) = \frac{\lambda_s\, e^{-\lambda_s \tau}}{g + \lambda_s} \left(1 - e^{-(g+\lambda_s)\, u}\right). \tag{S46}$$

Both components permit closed-form solutions: the Erlang component because k is an integer, and the exponential component by direct integration.

4 Model hazard functions

The primary model uses Hill functions for the antibiotic pressure:

$$h_S(C) = k_T \cdot k_{S/T} \cdot \frac{C^n}{K^n + C^n}, \tag{S47}$$

$$h_T(C) = k_T \cdot \frac{C^n}{K^n + C^n}, \tag{S48}$$

$$r_{S \to T}(C, a) = m(a) \left[r_0 + k_{ST} \cdot \frac{C^{n_{ST}}}{K_{ST}^{n_{ST}} + C^{n_{ST}}} \right]. \tag{S49}$$

The death hazards h_S and h_T share a common Hill coefficient n and half-maximal concentration K, differing only in their maximal rates: $\alpha_S = k_T \cdot k_{S/T}$ for susceptible cells and $\alpha_T = k_T$ for transiently tolerant cells. The constraint $k_{S/T} > 1$ ensures $h_S(C) > h_T(C)$ for all $C > 0$. Sharing n and K encodes the assumption that transient tolerance acts by reducing the maximal kill rate rather than shifting the dose–response curve, and reduces the number of free parameters.

Wake-rate constraint. We impose $\lambda_{\text{eff}}(a) \leq k_T$ for all culture ages in the dataset, where $\lambda_{\text{eff}}(a)$ is the effective lag rate defined in (S14). Since $h_T(C) \to k_T$ as $C \to \infty$, this constraint ensures that at saturating antibiotic concentrations, the transiently tolerant kill rate equals or exceeds the effective rate at which dormant cells wake into the susceptible pool. Without this constraint, the model could predict that T cells are generated (via $S \to T$ switching) faster than they are cleared at arbitrarily high concentrations, implying implausible persistence of the transiently tolerant class under conditions where we experimentally observe near-complete killing (Figure 4e, 250 μg/mL). With the constraint in place, high C and long τ efficiently clear both S and T cells, and the survivor pool contracts to the residual tail π_D, which itself becomes vanishingly small at treatment durations well beyond the mean lag time. The constraint is implemented as a quadratic penalty (see §5). Using λ_{eff} rather than the bare Erlang rate λ is critical when the mixture weight $w \approx 0$: in that regime, the Erlang rate can be very large while the actual wake rate is governed by the slow exponential component λ_s.

Two variants of the Hill model are compared:

1. **Hill-Hybrid** (primary, 13 free parameters): uses the full hybrid Erlang+Exponential lag distribution (S8) with both w and λ_s fitted. This is the production model (Table S3).
2. **Hill-Erlang** (11 free parameters): uses a pure Erlang lag distribution ($w = 1$ fixed, λ_s not fitted). This intermediate model tests whether the slow-exponential lag component improves fit quality.
3. Additionally, a reduced **Linear minimal** model variant was also fitted to the data for model comparison purposes (§5.4): a **Linear-Hazard minimal model** (7 parameters; hazards proportional to C without saturation, pure Erlang lag). The Linear minimal model is thus low-concentration approximation of the Hill model.

Table S3: Model parameters and their presence across model variants.

Symbol	Biological meaning	Fitted	Hill-Hybrid	Hill-Erlang	Linear
k	Erlang shape (number of lag stages)	Yes	✓	✓	✓
w	Erlang mixture weight	Yes	✓	–	–
λ_s	Slow-exit rate, exponential component (h^{-1})	Yes	✓	–	–
μ_0	Baseline mean lag time, Erlang component (h)	Yes	✓	✓	✓
$\Delta\mu_{24+}$	Additional mean lag per 24 h of starvation (h)	Yes	✓	✓	✓
k_T	Maximal kill rate, T state (h^{-1})	Yes	✓	✓	✓
$k_{S/T}$	Kill-rate ratio h_S/h_T (> 1)	Yes	✓	✓	✓
k_{ST}	Maximal $S \to T$ switching rate (h^{-1})	Yes	✓	✓	✓
K	Shared half-max. conc. for h_S, h_T ($\mu g\,mL^{-1}$)	Yes	✓	✓	–
K_{ST}	Half-max. conc. for $r_{S\to T}$ ($\mu g\,mL^{-1}$)	Yes	✓	✓	–
n	Shared Hill coefficient for h_S, h_T	Yes	✓	✓	–
n_{ST}	Hill coefficient for $r_{S\to T}$	Yes	✓	✓	–
a_{50}	Culture age at half-maximal stress history (h)	Yes	✓	✓	✓
r_0	Baseline $S \to T$ switching rate at $C = 0$ (h^{-1})	No	✓	✓	✓
g	Post-treatment balanced growth rate (h^{-1}); not fitted, as it does not enter the survival fraction predictions	Fixed	✓	✓	✓

5 Fitting Procedure and Sensitivity Analysis

5.1 Parameter estimation

All parameters were fitted by minimising a composite objective in log-space using L-BFGS-B (SciPy 1.14) with multistart optimisation (240 random initialisations, jitter $\sigma = 3.5$ in log-space). The composite objective is

$$\mathcal{L}(\theta) = \mathcal{L}_{\text{Dir}} + \lambda_{\text{surv}}\,\mathcal{L}_{\text{surv}} + \lambda_{\text{pen}}\,\mathcal{P}_{\text{tol}} + \mathcal{P}_{\text{hill}} + \mathcal{P}_{\text{regime}} + \lambda_{\text{reg}}\,\mathcal{P}_{\text{reg}}, \tag{S50}$$

where the six terms are described below. The Erlang shape k was selected by grid search over $k = 2, \dots, 14$, refitting all continuous parameters at each value of k. Parameter bounds are listed in Table S4.

Four-class Dirichlet NLL ($\mathcal{L}_{\text{Dir}}$). For each experimental condition j with observed fractions $\hat{p}_j = (\hat{p}_S, \hat{p}_T, \hat{p}_D, \hat{p}_{\text{dead}})_j$ and predicted fractions p_j:

$$\mathcal{L}_{\text{Dir}} = -\sum_j \sum_{c\in\{S,T,D,\text{dead}\}} w_c\big(\kappa\,\hat{p}_{j,c} - 1\big)\ln p_{j,c}, \tag{S51}$$

with Dirichlet concentration parameter $\kappa = 5000$ and optional class weights w_c.

Survivor-composition NLL ($\mathcal{L}_{\text{surv}}$). The four-class Dirichlet NLL is dominated by the dead class at high concentrations (often >99% dead), leaving almost no gradient signal about *which state* the survivors belong to. The survivor-composition NLL addresses this by operating on the renormalised survivor fractions:

$$\phi_c = \frac{p_c}{\sum_{c'} p_{c'}}, \qquad \hat{\phi}_c = \frac{\hat{p}_c}{\sum_{c'} \hat{p}_{c'}}, \qquad c, c' \in \{S, T, D\}, \tag{S52}$$

$$\mathcal{L}_{\text{surv}} = -\sum_j \sum_{c \in \{S,T,D\}} \left(\kappa_{\text{surv}}\, \hat{\phi}_{j,c} - 1\right) \ln \phi_{j,c}, \tag{S53}$$

with $\kappa_{\text{surv}} = 500$. Conditions with zero observed survivors are excluded. This term ensures that the model correctly predicts D-dominance among survivors at high C even though the total surviving fraction is vanishingly small.

Tolerance penalty ($\mathcal{P}_{\text{tol}}$). Enforces $h_T(C)/h_S(C) < \rho$ (default $\rho = 0.8$) across a concentration grid $C \in [0.05, 300]$ and representative ages, using a softplus barrier. With shared K and n, this ratio is the constant $1/k_{S/T}$, so the penalty reduces to enforcing $k_{S/T} > 1/\rho$.

Hill-lambda constraint ($\mathcal{P}_{\text{hill}}$). A quadratic penalty enforcing $\lambda_{\text{eff}}(a) \le k_T$ for all culture ages in the dataset:

$$\mathcal{P}_{\text{hill}} = \lambda_{\text{hill}} \cdot \max\!\Big(0,\ \max_a \big[\lambda_{\text{eff}}(a) - k_T\big]\Big)^2, \tag{S54}$$

where $\lambda_{\text{hill}} = 10^6$. This uses the effective lag rate $\lambda_{\text{eff}}(a)$ from (S14) rather than the bare Erlang rate, which is critical when $w \approx 0$.

Regime-dominance penalty ($\mathcal{P}_{\text{regime}}$). Encourages $k_T/\lambda_{\text{eff}}(a) \ge \beta_{\min}$ (default $\beta_{\min} = 2$), targeting the boundary where permanent T-over-D dominance becomes impossible at saturating C:

$$\mathcal{P}_{\text{regime}} = \frac{\lambda_{\text{regime}}}{|A|} \sum_{a \in A} \frac{1}{k_s} \ln\!\Big(1 + e^{k_s(\beta_{\min} - k_T/\lambda_{\text{eff}}(a))}\Big), \tag{S55}$$

with $\lambda_{\text{regime}} = 10^4$.

Parameter regularisation ($\mathcal{P}_{\text{reg}}$). Softplus penalties on biologically implausible parameter values: $n > 10$, $n_{ST} > 10$, $r_0 > 0.5$, and $k_{S/T} < 1.5$.

The default hyperparameters are $\lambda_{\text{surv}} = 1$, $\lambda_{\text{pen}} = 10^4$, and $\lambda_{\text{reg}} = 10^3$.

Table S4: Parameter bounds (natural scale). The Hill-Hybrid model fits all 13 parameters; the Hill-Erlang model fixes $w = 1$ and omits λ_s; the Linear model additionally omits K, K_{ST}, n, n_{ST} and fixes $n = n_{ST} = 1$.

Parameter	Lower bound	Upper bound
w	0	1
λ_s	10^{-6}	10
μ_0	0.03	400
$\Delta\mu_{24+}$	0	400
$k_{S/T}$	1.0	1000
k_T	10^{-4}	100
k_{ST}	10^{-3}	1000
K	10^{-6}	10^3
K_{ST}	10^{-6}	10^6
n	0	4.5
n_{ST}	0	4.5
a_{50}	0	3×10^5

Table S5: Maximum-likelihood parameter estimates for the Hill-Hybrid model ($k = 6$, 13 free parameters). The Erlang shape k was selected by grid search; all other parameters were fitted by L-BFGS-B multistart optimisation.

Symbol	Parameter	MLE
k	Erlang shape	6
w	Erlang mixture weight	0.999
λ_s	Slow-exit rate (h^{-1})	1.63×10^{-4}
μ_0	Baseline mean lag (h)	0.715
$\Delta\mu_{24+}$	Additional mean lag per 24 h (h)	0.184
k_T	Max. kill rate, T (h^{-1})	3.55
$k_{S/T}$	Kill-rate ratio h_S/h_T	2.26
k_{ST}	Max. switching rate (h^{-1})	1.74
K	Half-max. conc., h_S/h_T (μg mL^{-1})	65.7
K_{ST}	Half-max. conc., $r_{S\to T}$ (μg mL^{-1})	32.6
n	Hill coefficient, h_S/h_T	1.19
n_{ST}	Hill coefficient, $r_{S\to T}$	2.36
a_{50}	Half-max. stress history (h)	26.0
r_0	Baseline switching rate (h^{-1})	0.0608

5.2 Sensitivity analysis

Parameter uncertainty was assessed by Laplace approximation at the MLE: the Hessian of the negative log-likelihood was computed by central finite differences, inverted to obtain an approximate covariance matrix in log-space, and used to generate 5 000 multivariate normal draws. These draws were propagated

through the model to obtain 95% prediction intervals on all survivor fractions (reported on the predicted-versus-observed plots, Figure 8a–d). The resulting marginal posterior distributions for all 13 Hill-Hybrid parameters are shown in Figure S11.

To assess whether the predicted survivor composition is robust to parameter uncertainty, we propagated the posterior draws through the model. Posterior histograms of the survivor fractions φ_T, φ_S, and φ_D at six representative treatment conditions are shown in Figures S12-S14. The corresponding one-dimensional composition envelopes-MLE curves with 95% posterior bands for all three fractions as a function of concentration C and treatment duration τ—are shown in Figure S15.

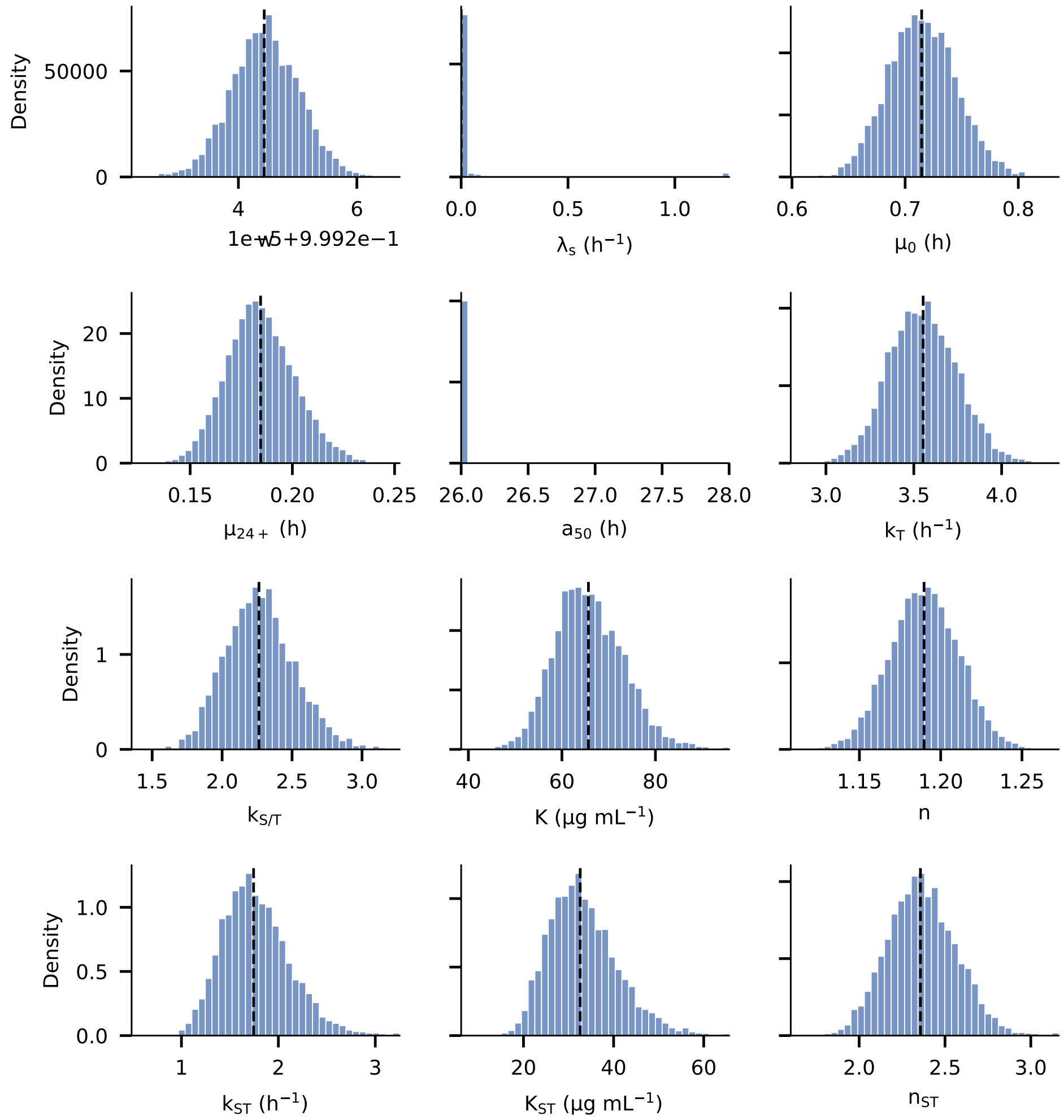

Figure S11: Marginal posterior distributions for all 13 Hill-Hybrid model parameters, obtained from the Laplace approximation (5 000 multivariate normal draws in log-space, transformed to natural scale). Dashed vertical lines indicate the maximum-likelihood estimates.

5.3 Fit quality for the lag-based survivor class

The model accurately reproduces the observed fractions of exposure-limited survivors ($X \subset S$), transiently tolerant survivors (T), and dead cells across the full experimental range (Figure 8a–c). The lag-based survivor fraction ($P \subset D$, Figure 8d) shows a systematic underprediction, which we discuss here.

Origin of the deviation. The predicted lag-based survivor fraction $\pi_D(\tau, a) = S_L(\tau; a)$ depends only on the lag-time distribution and the treatment duration; it is independent of antibiotic concentration (Equation S26). For a given (τ, a), the model therefore predicts a single value of π_D regardless of C. The observed fractions, however, represent at most 2–14 cells per condition (out of >10 000 cells per condition), placing them at the limit of reliable frequency estimation. Small-number fluctuations in this regime can produce apparent deviations of an order of magnitude or more. In addition, the Erlang distribution, while well suited to capturing the bulk of the resuscitation dynamics, has exponentially decaying tails. The real lag-time distribution likely has a heavier tail, consistent with the observation that a small number of cells with exceptionally long lag times persist even at $\tau = 8$ h, where the Erlang survival function is negligible ($S_L(8;\ a{=}24) \approx 10^{-14}$).

Classification boundary at short treatment durations. The model classifies any cell with $L > \tau$ as a lag-based survivor, without imposing a minimum lag duration for what constitutes a "true" persister. At short treatment durations ($\tau < {\sim}1$ h), a substantial fraction of the population has lag times exceeding the brief treatment window—these are ordinary slow-waking cells, not the rare, deeply dormant persisters that survive prolonged high-dose exposure. This accounts for the model predicting a high lag-based survivor fraction at short τ across all concentrations (visible as the blue band in Figure 8f). Experimentally, subsequent growth dynamics distinguish these cells from deep persisters, but the model's $L > \tau$ criterion does not make this distinction.

Impact on conclusions. The underprediction of the persister fraction does not affect the model's central predictions for two reasons. First, the post-treatment regrowth analysis (§3) shows that persisters contribute to regrowth only after completing their residual lag ($L_{\text{res}} = L - \tau$), which introduces a substantial delay relative to T and $X \subset S$ survivors that resume growth immediately. Even if the true persister fraction were an order of magnitude higher than predicted, the growth-discount penalty (Equation S42) ensures that T-descended cells still dominate early regrowth under clinically relevant conditions. Second, the conditions where persisters are most underpredicted (high C, long τ) are precisely those where near-complete killing is observed (> 99.9% dead), so the absolute number of survivors of any class is very small.

5.4 Model comparison

The three model variants defined in §4 were fitted to the same dataset ($n = 10$ conditions) and compared using the four-class Dirichlet NLL ($\kappa = 5000$), AIC, and BIC (Table S6).

Table S6: Model comparison (lower is better for all criteria). Δ values are relative to the Hill-Hybrid model.

Model	Free params (k)	NLL	AIC	BIC
Hill-Hybrid (primary)	13	15 208.6	30 443.1	30 447.1
Hill-Erlang	11	15 275.7	30 573.4	30 576.8
Linear (minimal)	7	15 957.5	31 929.1	31 931.2

Both AIC and BIC favour the Hill-Hybrid model, indicating that the additional parameters are supported by the data rather than reflecting overfitting. Critically, the central prediction, that transiently tolerant-descended cells dominate post-treatment regrowth under clinically relevant conditions, is reproduced by all three model variants, confirming that this conclusion is robust to model specification and does not depend on the specific parametric form of the hazard functions. The models diverge at high antibiotic concentrations ($C \geq 50\,\mu\text{g}\,\text{mL}^{-1}$), where the reduced models cannot capture dose–response saturation, and in the persister tail, where the hybrid lag distribution better reproduces the observed frequency of late-waking cells.

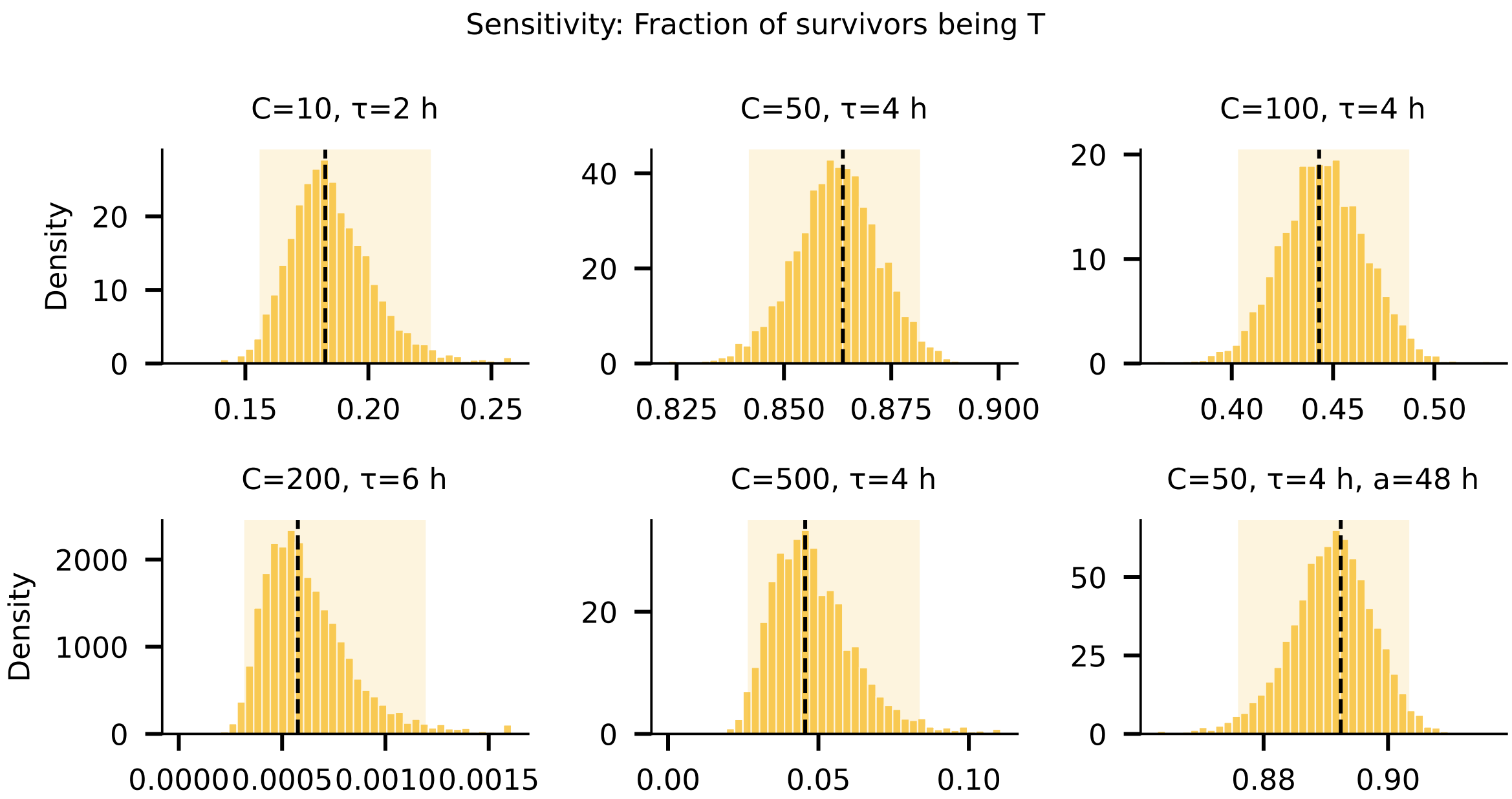

Figure S12: Sensitivity analysis: fraction of survivors being tolerant. Posterior histograms of the tolerant survivor fraction $\pi_T/(\pi_S + \pi_T + \pi_D)$ at six representative (C, τ, a) conditions, computed from 5 000 Laplace-approximation draws. Dashed lines indicate the MLE; shaded bands mark the 95% confidence interval.

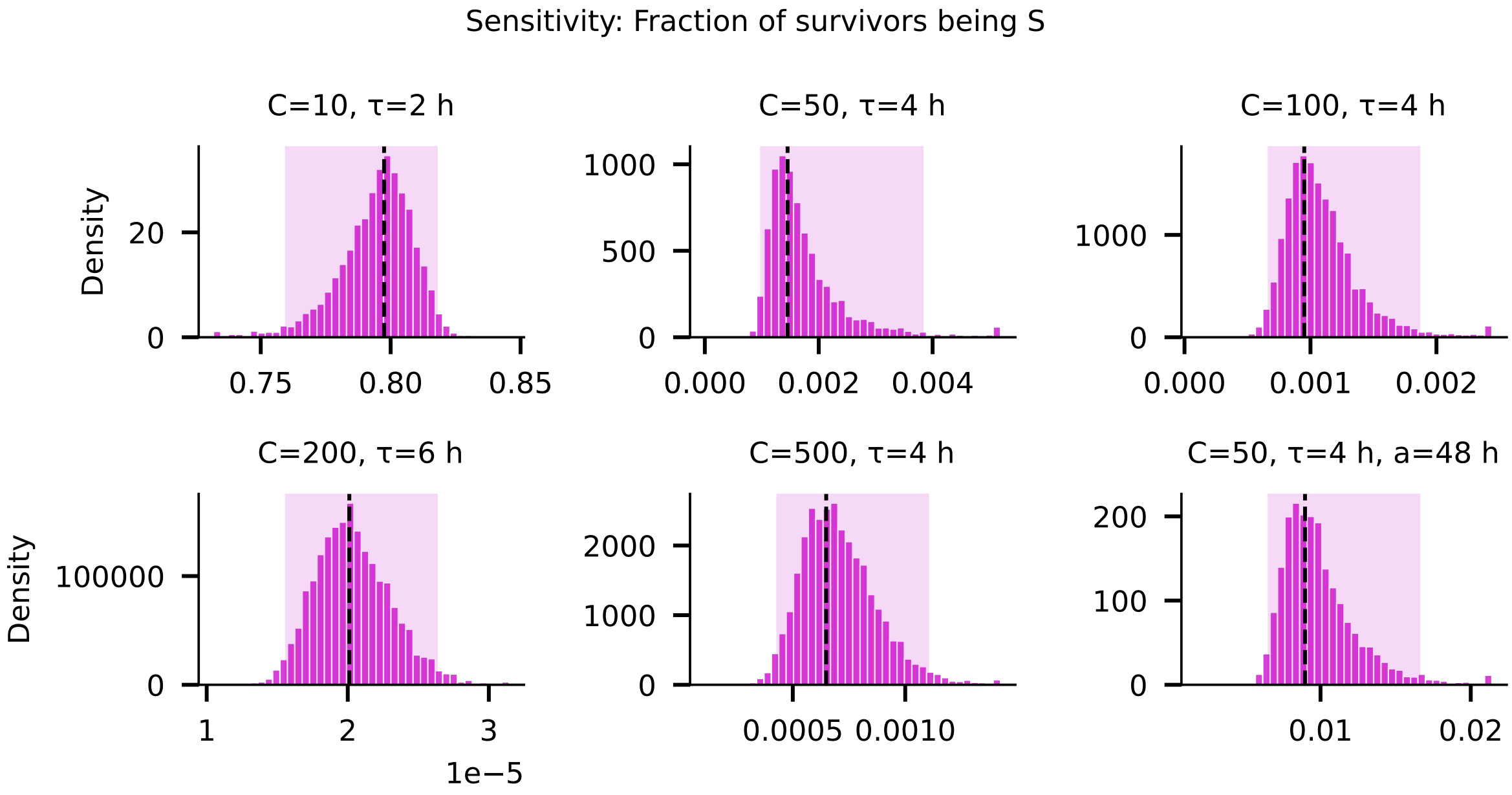

Figure S13: Sensitivity analysis: fraction of survivors being susceptible. Posterior histograms of the exposure-limited survivor fraction $\pi_S/(\pi_S + \pi_T + \pi_D)$ at the same six conditions as Figure S12.

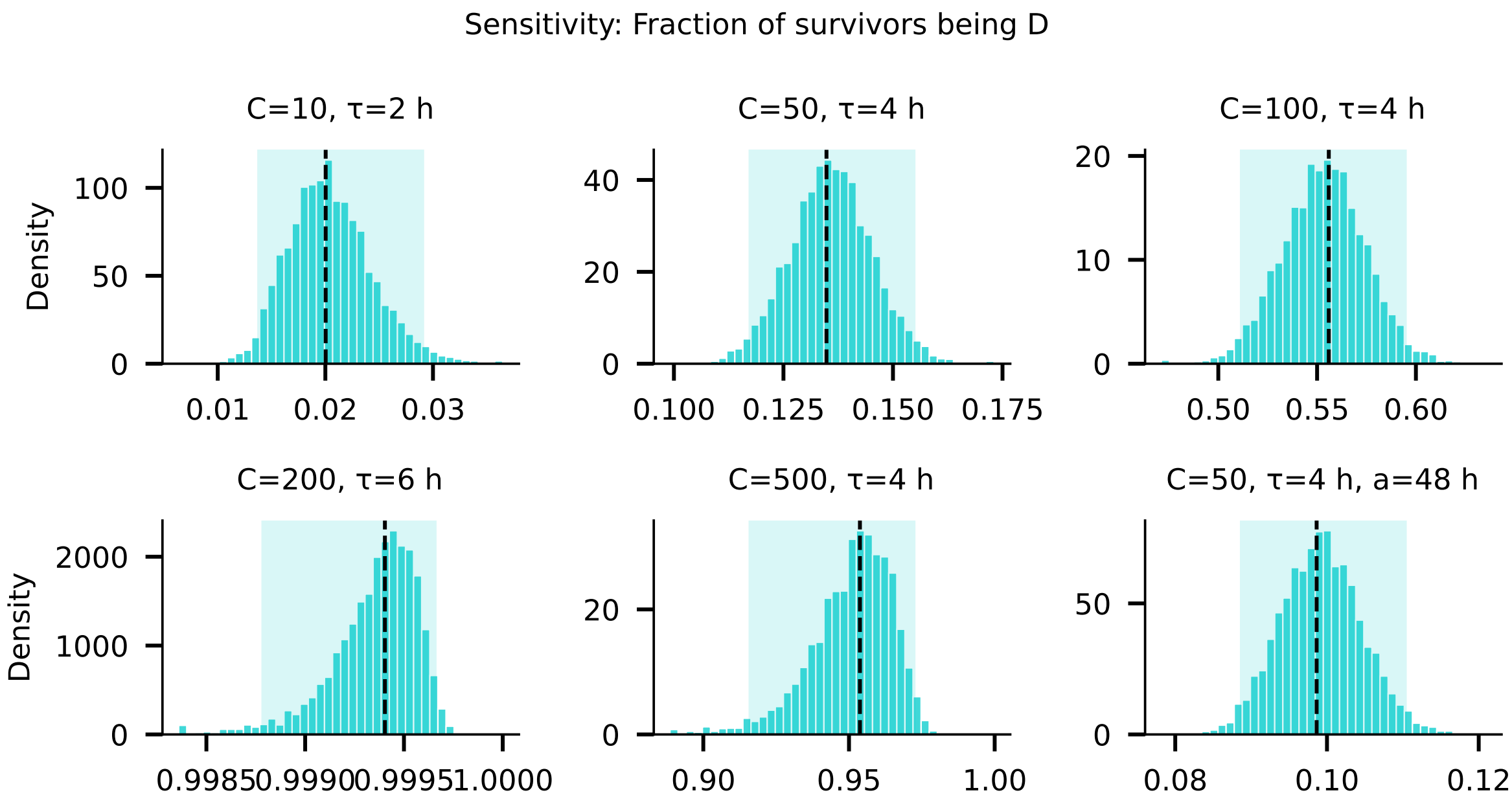

Figure S14: Sensitivity analysis: fraction of survivors being persisters. Posterior histograms of the lag-based (persister) survivor fraction $\pi_D/(\pi_S + \pi_T + \pi_D)$ at the same six conditions as Figure S12.

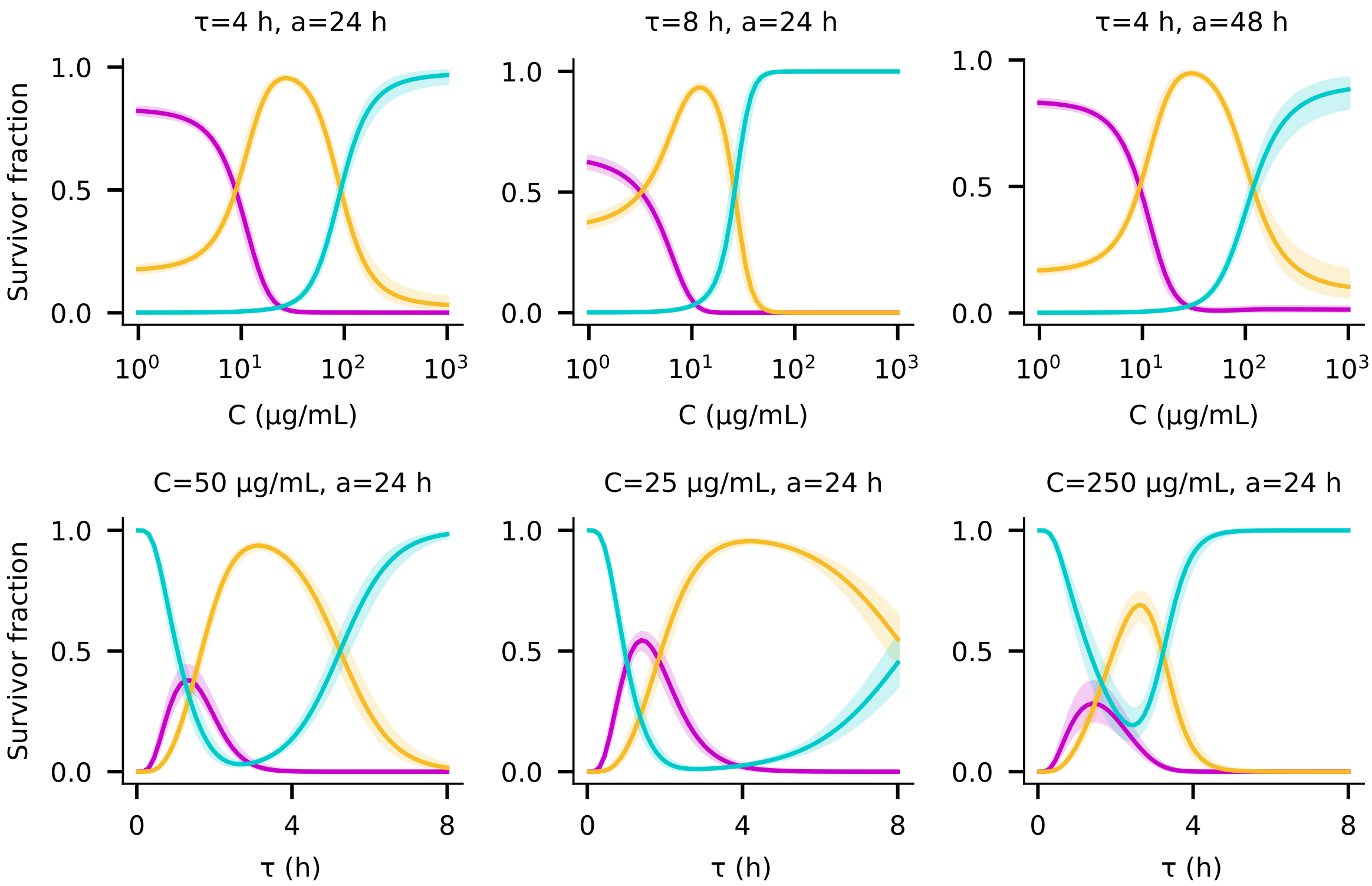

Figure S15: Posterior composition envelopes. MLE survivor-fraction curves (solid) with 95% posterior bands (shaded) for all three survivor classes. Top row: composition vs. antibiotic concentration C at three (τ, a) slices. Bottom row: composition vs. treatment duration τ at three (C, a) slices.

References

1. Basan, M., Honda, T., Christodoulou, D., *et al.* A Universal Trade-off between Growth and Lag in Fluctuating Environments. *Nature* **584,** 470–474 (2020).

2. Joynt, G. M., Lipman, J., Gomersall, C. D., Young, R. J., Wong, E. L. Y. & Gin, T. The Pharmacokinetics of Once-Daily Dosing of Ceftriaxone in Critically Ill Patients. *Journal of Antimicrobial Chemotherapy* **47,** 421–429 (2001).

3. *Ceftriaxone: Rationale for EUCAST Clinical Breakpoints* tech. rep. 1 (EUCAST, 2023), 4.

4. Balaban, N. Q., Helaine, S., Lewis, K., *et al.* Definitions and Guidelines for Research on Antibiotic Persistence. *Nature Reviews Microbiology* **17,** 441–448 (2019).

5. Fanous, J., Claudi, B., Tripathi, V., Li, J., Goormaghtigh, F. & Bumann, D. Limited Impact of Salmonella Stress and Persisters on Antibiotic Clearance. *Nature* **639,** 181–189 (2025).

6. Lichtner, G. *Glichtner/Pystackreg* 2026.

7. Miles, A., Kirkham, J., Durant, M., *et al. Zarr-Developers/Zarr-Python: V2.4.0* Zenodo. 2020.

8. Hardo, G. *Georgeoshardo/PyMMM* 2025.

9. Cutler, K. J., Stringer, C., Lo, T. W., *et al.* Omnipose: A High-Precision Morphology-Independent Solution for Bacterial Cell Segmentation. *Nature Methods* **19,** 1438–1448 (2022).

10. Hardo, G., Noka, M. & Bakshi, S. Synthetic Micrographs of Bacteria (SyMBac) Allows Accurate Segmentation of Bacterial Cells Using Deep Neural Networks. *BMC Biology* **20,** 263 (2022).

11. Van der Walt, S., Schönberger, J. L., Nunez-Iglesias, J., *et al.* Scikit-Image: Image Processing in Python. *PeerJ* **2,** e453 (2014).

12. Virtanen, P., Gommers, R., Oliphant, T. E., *et al.* SciPy 1.0: Fundamental Algorithms for Scientific Computing in Python. *Nature Methods* **17,** 261–272 (2020).

13. Sofroniew, N., Lambert, T., Bokota, G., *et al. Napari: A Multi-Dimensional Image Viewer for Python* Zenodo. 2024.
